# Implementation of Enhance®Fitness: a scoping review using the RE-AIM framework

**DOI:** 10.1093/geront/gnag102

**Published:** 2026-05-09

**Authors:** Wenting Peng, Sarah McKiddy, Michiyo Tomioka, Dina L Maruca, Nancy M Gell, Matthew L Smith, Caitlin Maloy, Basia Belza

**Affiliations:** School of Nursing, University of Washington, Seattle, Washington, United States; School of Nursing, University of Washington, Seattle, Washington, United States; Center on the Family, University of Hawaii, Honolulu, Hawaii, United States; Department of Orthopaedics and Division of Physical Therapy, West Virginia University, Morgantown, West Virginia, United States; Rehabilitation and Movement Science, University of Vermont, Burlington, Vermont, United States; Department of Applied Health Science, School of Public Health, Indiana University, Bloomington, Indiana, United States; Center for Health by Design, School of Public Health, Indiana University, Bloomington, Indiana, United States; Health Sciences Library, University of Washington, Seattle, Washington, United States; School of Nursing, University of Washington, Seattle, Washington, United States

**Keywords:** Evidence-based programs, Older adults, Physical activity, RE-AIM, Implementation science

## Abstract

**Background and Objectives:**

Enhance®Fitness (EF) is an evidence-based group exercise program shown to promote physical and mental health among older adults. The scoping review aims to systematically map and synthesize the literature on EF using the RE-AIM framework (Reach, Effectiveness, Adoption, Implementation, and Maintenance).

**Research Design and Methods:**

We conducted a scoping review of implementation studies focusing on EF or its predecessor, the Lifetime Fitness Program. Searches were conducted in 6 databases (PubMed, Embase, CINAHL, SCOPUS, Web of Science, and PsycINFO) and supplemented with EF-related publications listed on the program’s official website. The search included all publications from January 1, 1980 through July 1, 2025.

**Results:**

We included 33 publications. Reach showed EF primarily engaged community-dwelling older adults, typically female. Effectiveness outcomes were generally positive, including improved physical and mental health and reduced health care utilization. Adoption occurred across varied settings, supported by trained instructors and organizational alignment. Implementation typically followed the standard EF protocol of three 60-min sessions per week, delivered in person or remotely. Facilitators included instructor training, organizational support, and social engagement, while barriers included attendance challenges, resource needs, and technology issues. Maintenance was demonstrated through sustained program delivery across diverse community settings, and participant-level maintenance was reflected in follow-up assessments of health outcomes at common time points of 4, 6, or 12 months.

**Discussion and Implications:**

EF is a widely adopted program with demonstrated benefits for older adults. Greater attention to equity and long-term sustainability is needed to strengthen future dissemination and support healthy aging.

Regular physical activity is widely recognized as a protective factor for older adults to promote physical and cognitive function and decrease the risk of all-cause mortality (Watts et al., 2022). Despite these benefits, physical inactivity is common among older adults. In 2022, 36.4% of U.S. adults aged 60 years and older worldwide did not meet the World Health Organization (WHO)–recommended physical activity levels (Strain et al., 2024). A systematic review suggested that key barriers to being physically active include poor motivation, declining health status, fear of falling, and environmental obstacles (Kilgour et al., 2024). These challenges are often magnified for people living with dementia, who on average are more sedentary and less active than cognitively healthy peers, reflecting broader gaps in accessibility, inclusion, and program fit (e.g., need for clear cues, sensory-friendly environments, and care-partner supports) (Di Lorito et al., 2023). Therefore, community-based, evidence-based group exercise programs are important to overcome these barriers, and promote safe, structured, and inclusive opportunities for physical activity among older adults, including those living with cognitive impairment.

A substantial body of peer-reviewed evidence supports the effectiveness of community-based, multicomponent exercise programs for older adults (Wang et al., 2026). Systematic and meta-analytic reviews have demonstrated that structured group exercise interventions reduce falls among community-dwelling older adults (Sherrington et al., 2020) and improve physical function and overall health status among pre-frail populations (Lim et al., 2024). These findings underscore the broader evidence base for exercise programs integrating aerobic, strength, balance, and flexibility components. However, prior reviews have primarily focused on intervention effectiveness across heterogeneous program models, with less attention to the implementation, dissemination, and long-term sustainability of specific, nationally scaled programs. Addressing these gaps requires an implementation perspective that considers how programs are delivered, adopted, and sustained in real-world settings.

Enhance®Fitness (EF), originally developed as the Lifetime Fitness Program (LFP), is a widely disseminated, evidence-based group exercise and falls-prevention program for older adults (Smith et al., 2014). Standard EF classes are delivered three times per week in 60-min sessions over a 16-week period, including aerobic, strength, balance, and flexibility exercises led by nationally-certified instructors (Sound Generations, 2025). The program operates across diverse community settings, including senior centers, YMCAs, health care systems, residential facilities, and faith-based organizations (Sound Generations, 2025). EF currently offers approximately 1,600 classes across more than 800 sites in 44 states and the District of Columbia, engaging over 120,800 older adults since 2001 (P. Denison, Sound Generations, personal communication, 2026). Previous studies have demonstrated that EF improves physical function (Fishleder et al., 2019), reduces falls (Batra et al., 2019), increases social engagement (Mays et al., 2021), and lowers health care utilization and costs (Nguyen et al., 2007). Despite its demonstrated effectiveness and broad reach, the literature describing EF’s implementation, adaptations, and long-term sustainability remains fragmented, limiting a full understanding of its overall public health impact. Petrescu-Prahova et al. (2017) conducted a scoping review summarizing EF dissemination and implementation evidence published between 2010 and 2015. However, with nearly a decade of additional research now available, an updated synthesis is needed. In particular, newer studies include those published after 2020, studies of remotely delivered EF (tele-EF), and work that more explicitly considers issues of access and equity. These additions provide a more current understanding about EF’s implementation and impact. This updated synthesis will require a broad implementation framework that can capture more recent innovations (e.g., remote delivery) and issues (e.g., equity).

Although EF incorporates exercise components common to many community-based physical activity programs for older adults (e.g., aerobic, strength, balance, and flexibility training), it is distinguished by several structural and implementation features. EF operates under a standardized, manualized curriculum supported by nationally certified instructors, fidelity monitoring tools, and a centralized data collection platform that prospectively tracks participant outcomes across sites. In addition, EF is embedded within a long-standing national dissemination network spanning diverse community organizations. These characteristics position EF not only as an evidence-based exercise program, but also as a mature implementation platform with sustained delivery at scale. Examining EF through an implementation science lens may therefore provide insights that extend beyond the program itself to inform broader dissemination of community-based physical activity interventions.

The RE-AIM Framework—comprising Reach, Effectiveness, Adoption, Implementation, and Maintenance—provides a methodical approach for evaluating the public health impact of interventions (Glasgow et al., 2019). RE-AIM is widely used in implementation and aging research because it moves beyond clinical effectiveness to examine how programs are delivered, disseminated, and sustained in real-world settings (Glasgow et al., 2019). Applying RE-AIM to EF provides an opportunity to synthesize evidence across multiple domains, highlight facilitators and barriers to program delivery, and assess long-term sustainability. Thus, the purpose of this scoping review was to: (a) systematically map and synthesize the literature on EF using the RE-AIM Framework; and (b) identify gaps and opportunities to strengthen EF’s dissemination, equity, and impact on healthy aging.

## Method

We conducted a scoping review to systematically map and synthesize the existing literature on the public health impact of EF. A scoping review is particularly suited for mapping the extent, range, and nature of evidence in a field and identifying gaps to guide future research (Peters et al., 2020). This review adhered to the Preferred Reporting Items for Systematic reviews and Meta-Analyses extension for Scoping Reviews (PRISMA-ScR) (see Supplementary Appendix 1) and was registered on Open Science Framework (https://doi.org/10.17605/OSF.IO/PCFDX).

### Eligibility criteria

Publications were eligible if they: (a) investigated EF or its predecessor, the LFP, as the primary focus; (b) reported primary data using quantitative, qualitative, or mixed methods designs; and (c) were published in the English language. We excluded reviews, editorials, conference abstracts, dissertations, and studies that did not explicitly evaluate EF or LFP. We excluded gray literature from this review because our objective was to systematically compare studies using RE-AIM, and inclusion of non–peer-reviewed sources would have introduced greater variability in reporting rigor and completeness. Comprehensiveness was supported by our supplementary search of EF’s official program website, which provided an additional source of academic publications beyond the database searches.

### Information sources and search strategy

A comprehensive search strategy was developed in collaboration with a health sciences librarian (CM). Six databases were searched, which included PubMed, CINAHL (EBSCOhost), Embase, Web of Science, PsycINFO (EBSCOhost), and the Cochrane Library. The strategy included all known names and abbreviations for EF and LFP. Our literature searching strategy aimed to balance sensitivity and specificity by carefully combining controlled vocabulary terms with restricted keywords and synonyms, and by limiting them to search results where those terms appeared in either the titles or the abstracts of articles. This strategy enabled us to identify results highly likely to be relevant while excluding articles that did not meet the inclusion criteria. The initial search was conducted on May 29, 2025, covering publications from January 1, 1980, to the search date. An updated search was performed on July 10, 2025, to identify additional publications through July 1, 2025. Therefore, the date range for the search was from January 1, 1980, to July 1, 2025. The PubMed search was iteratively developed using keywords, synonyms, and MeSH terms with Boolean operators, and was translated into the other databases using controlled vocabulary and database-specific syntax. The full search strategies for all six databases are provided in Supplementary Appendix 2. To ensure completeness, we also included publications listed on the official EF program website (https://projectenhance.org/what-is-an-evidence-based-program/citations/). Records from both the database searches and the EF website were imported into Covidence (https://www.covidence.org/) for deduplication and screening.

### Screening and data extraction

**Figure 1:**
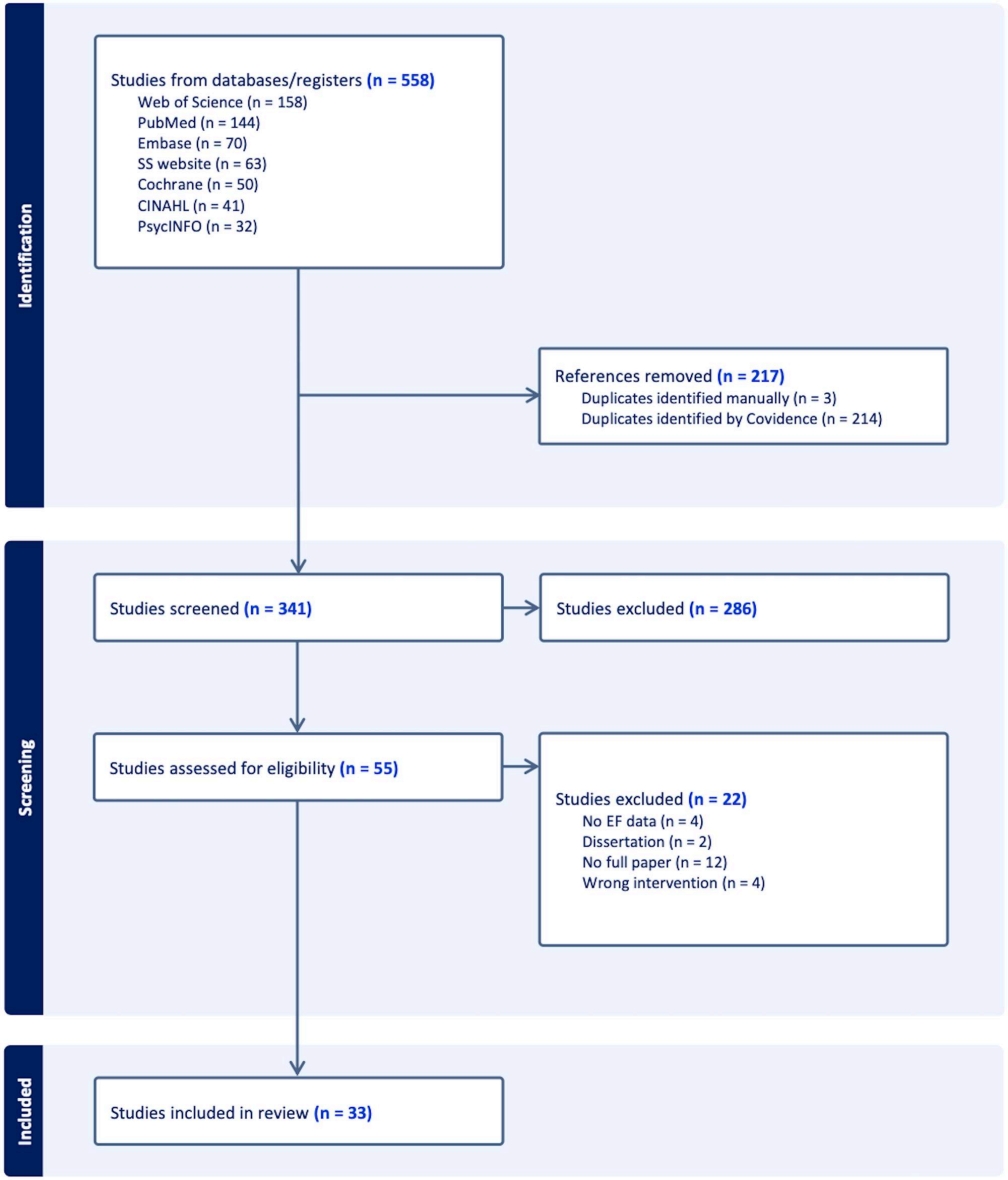
Flow diagram of study selection for the Enhance^®^Fitness scoping review. PRISMA flow diagram showing 558 records identified, 217 duplicates removed, 341 records screened, 55 full-text articles assessed, 22 excluded, and 33 studies included in the review.

Two reviewers (WP and BB) screened titles and abstracts, followed by full texts, against the eligibility criteria. Reasons for exclusion were documented in covidence, and results are presented in the PRISMA-ScR flow diagram (Figure 1). The data extraction form was developed collaboratively by three authors (WP, SM, and BB), pilot-tested on one study, and refined in consultation with the review team. The final form was structured around the RE-AIM framework and included study characteristics (e.g., year, design, setting, sample size, target population), as well as information corresponding to each RE-AIM domain: Reach (e.g., demographics, chronic conditions, recruitment); Effectiveness (e.g., outcomes, adherence, adverse events, health care utilization); Adoption (e.g., setting type, organizational characteristics, instructor training); Implementation (e.g., frequency and duration of sessions, delivery mode, fidelity, barriers, facilitators); and Maintenance (e.g., long-term outcomes, continued delivery, financial sustainability). Two reviewers independently abstracted data for each study and reconciled differences within Covidence. Disagreements were resolved through discussion and consensus between the two reviewers; when consensus could not be reached, a third reviewer was consulted to make the final determination. The study limitations and equity-related variables were captured. The final dataset was exported into Excel to facilitate synthesis. To assess potential redundancy, we examined study characteristics (e.g., data sources, samples, time frames, and analytic approaches) to identify possible overlap across publications. When overlapping cohorts were identified, we retained only the most comprehensive report if outcomes were duplicative; however, reports contributing distinct information across RE-AIM domains were included. During the analysis phase, we used the Consolidated Framework for Implementation Research (CFIR) to further organize and interpret the reported barriers and facilitators to EF implementation (Damschroder et al., 2022). While RE-AIM guided the overall structure of the synthesis, CFIR was used as a complementary framework to provide a detailed categorization of implementation-related factors. Specifically, implementation-related findings were grouped into CFIR domains, including intervention characteristics, inner and outer setting factors, individual-level characteristics, and implementation processes. Additionally, because some included publications were co-authored by members of this review team, those authors did not assess their own studies to minimize potential bias.

## Results

### Study selection

We identified 558 records (Web of Science, *n* = 158; PubMed, *n* = 144; Embase, *n* = 70; EF program website, *n* = 63; Cochrane, *n* = 50; CINAHL, *n* = 41; PsycINFO, *n* = 32). After removing 217 duplicates (3 manually and 214 through Covidence), 341 records remained for title and abstract screening. Screening excluded 286 records, leaving 55 articles for full-text assessment. Of these, 22 studies were excluded for the following reasons: no full paper available (*n*  = 12), no data about EF (*n*  = 4), intervention was not EF (*n*  = 4), or dissertation (*n*  = 2). Ultimately, 33 studies were included in the review (Figure 1). All 33 included studies were identified through the database search and screening process. As a supplementary check, we reviewed the EF program website, where 28 of the included studies were also listed.

### Study characteristics

Table 1 presents the characteristics of the identified 33 studies. The majority were published between 2010 and 2019 (54.5%), with a growing proportion from 2020 onward (27.3%). Prospective (cohort or longitudinal) and quasi-experimental designs were the most common, accounting for nearly two-thirds of the included studies. Most studies were conducted in community settings (81.8%), with a smaller subset delivered in clinical or home settings (with remotely-delivered EF). Additional details for each study are provided in Table S1 in Supplementary Appendix 3.

**Table 1:** Study characteristics (*N* = 33).

Characteristics	*N* (%)
**Published year**	
Before 2000	1 (3.0)
2000–2009	5 (15.2)
2010–2019	18 (54.5
2020–2025	9 (27.3)
**Study design**	
Qualitative	6 (18.2)
Cross-sectional	5 (15.2)
Retrospective (case-control, historical prospective)	4 (12.1)
Prospective (cohort, longitudinal)	12 (36.4)
Quasi-experimental	9 (27.3)
Randomized controlled trial	3 (9.1)
Mixed methods	4 (12.1)
**Setting**	
Community setting	27 (81.8)
Clinical setting	5 (15.2)
Home	5 (15.2)
Other	3 (9.1)

*Note*. Percentages exceed 100% due to some studies reporting multiple designs and settings.

### RE-AIM domains

#### Reach

Of the 33 included studies, 29 focused on EF participants, 4 focused on program staff, coordinators, and associations. Across the 29 participant-focused studies, EF has engaged a racially and ethnically diverse population of older adults, including American Indian/Alaska Native, Asian/Asian American, Black/African American, Native Hawaiian/Pacific Islander, and Hispanic/Latino participants (Table S2, Supplementary Appendix 3). While many studies reported the proportion of female participants, none reported data on specific gender identity or sexual orientation. Most studies targeted community-dwelling older adults, although some targeted specific subgroups such as Medicare enrollees, ethnically diverse populations, rural cancer survivors, sedentary or low-active adults with self-reported physician-diagnosed arthritis, older adults with diabetes, rural residents with symptomatic knee osteoarthritis, older Korean immigrants, and rural community-dwelling older adults on Kaua‘i, Hawai‘i. Common inclusion criteria included minimum age thresholds (≥60 or ≥65 years), ability to ambulate independently, and being new to EF. Exclusion criteria varied but often related to serious medical conditions or functional limitations that could hinder participation. Recruitment strategies ranged from existing client databases to community outreach methods such as flyers, posters, and word of mouth. Fishleder et al. (2019) used a more comprehensive approach by developing a recruitment toolkit that included brochures, posters, letters to patients identified through electronic health records, and content for print and social media, which was implemented in partnership with a community health organization (details for each study are included in Table S2, Supplementary Appendix 3).

In addition to demographic and recruitment characteristics, some studies described participants’ chronic conditions (Table 2). Of the 33 studies, 22 (66.7%) reported that their EF participants had at least one chronic condition. The most frequently reported conditions were arthritis (15 studies, 45.5%), hypertension (14 studies, 42.4%), diabetes (13 studies, 39.4%), and heart disease (10 studies, 30.3%). Less commonly reported were cancer (6 studies, 18.2%), chronic obstructive pulmonary disease (3 studies, 9.1%), stroke (1 study, 3.0%), heart attack (1 study, 3.0%), chronic kidney disease (1 study, 3.0%), and dementia (1 study, 3.0%). Mental health conditions were also represented: depression was reported in 6 studies (18.2%), sometimes in combination with anxiety, post-traumatic stress disorder, or bipolar disorder. People living with dementia were excluded in 7 studies (21.2%).

**Table 2:** Reporting of chronic conditions across Enhance^®^Fitness studies.

Study	Heart attack	Heart disease	Hypertension	Arthritis	Diabetes	Cancer	Chronic kidney disease	Chronic obstructive pulmonary disease	Stroke	Dementia	Other
Ackermann et al. (2003)											Chronic disease score
Ackermann et al. (2008)		X		X	X						High-density lipoprotein cholesterol, triglycerides, hemoglobin A1C, RxRisk comorbidity score
Agmon et al. (2015)		X	X	X	X					O	Depression
Batra et al. (2016)											Number of chronic conditions
Batra et al. (2019)											Depression, number of chronic conditions
Fishleder et al. (2019)		X	X	X	X	X		X			Depression
Gell et al. (2021)										O	Symptomatic knee osteoarthritis; number of medical conditions
Gell et al. (2023)										O	Number of medical conditions
Gell et al. (2024)						X					
Greenwood-Hickman et al. (2015)		X	X	X	X					X	Walking disorder, osteoporosis, musculoskeletal conditions, Visual impairment
Jones et al. (2024)			X	X	X						Obesity
Kohn et al. (2015)			X	X	X						
Mays et al. (2021)										O	Number of chronic conditions
Nguyen et al. (2007)		X	X	X	X						Depression
Patel et al. (2022)				X						O	Obesity
Rosenberg et al. (2014)		X	X	X	X	X					Obesity
Sin et al. (2005)		X	X	X	X						
Smith et al. (2014)		X	X	X	X	X					Lung disease
Steinman et al. (2024)		X	X	X	X	X	X	X	X	O	Depression, anxiety, PTSD, bipolar disorder, digestive problems, osteoarthritis
Sugihara et al. (2011)			X	X	X						
Tomioka et al. (2012)	X	X	X	X	X	X		X			Depression
Tomioka et al. (2019)			X	X							

*Note*. X indicates that the study reported the condition among participants; O indicates that the condition was used as an exclusion criterion; PTSD = post-traumatic stress disorder.

#### Effectiveness

Across the included studies, EF was evaluated on diverse outcomes including physical function, psychological well-being, health care utilization, and other health indicators (Table 3). Statistically significant improvements were reported for most outcomes, except for blood pressure. Physical function was the most frequently assessed, with 15 studies reporting outcomes such as chair stands, arm curls, 8-foot up-and-go, dual-task walking performance, single-leg balance, composite physical function scores, daily steps, exercise intensity, and frequency of 30-min exercise duration. Psychological outcomes were examined in five studies and included loneliness, depression, social isolation, social interaction, sleep quality, and technology-related anxiety. Health care utilization and cost were reported in three studies, covering hospitalizations, inpatient stays, primary and specialty care visits, diabetes-related visits, and total health care costs. Other outcomes were reported in eight studies, including self-rated health status, number of falls, falls requiring medical treatment, arthritis pain intensity, quality of life (SF-36, SF-12, Medical Outcomes Study Short Form), and participant satisfaction. Blood pressure was assessed in one study and did not show statistically significant improvement.

**Table 3:** Effectiveness.

Outcomes	Study
**Physical function**	
Dual-task walking performance	Agmon et al. (2015)
Chair stands	Batra et al. (2019); Fishleder et al. (2019); Gell et al. (2024); Jones et al. (2024); Kohn et al. (2015); Palmer et al. (2016); Sin et al. (2005); Tomioka et al. (2012, 2019)
Arm curl	Batra et al. (2019); Fishleder et al. (2019); Jones et al. (2024); Kohn et al. (2015); Palmer et al. (2016); Sin et al. (2005); Tomioka et al. (2012, 2019)
8-foot up-and-go	Batra et al. (2019); Fishleder et al. (2019); Jones et al. (2024); Kohn et al. (2015); Palmer et al. (2016); Sin et al. (2005); Tomioka et al. (2012, 2019)
Composite physical function score	Fishleder et al. (2019); Jones et al. (2024)
Self-reported restricted-activity days	Wallace et al. (1998)
Energy/fatigue	Steinman et al. (2024)
Frequency of daily exercise of at least 30-min duration	Palmer et al. (2016)
Physical benefits (qualitative)	Kohn et al. (2016)
Single-leg stand balance	Gell et al. (2024) ^a^
Steps per day	Gell et al. (2021)
Exercise intensity	Gell et al. (2023)
PROMIS physical function short form	Gell et al. (2024)
2-min step test	Jones et al. (2024)
**Psychological function**	
Loneliness	Mays et al. (2021); Steinman et al. (2024)
Depression	Steinman et al. (2024); Wallace et al. (1998)
Sleep quality	Steinman et al. (2024)
Technology anxiety	Steinman et al. (2024)
Social isolation	Mays et al. (2021)
Social interaction (qualitative)	Kohn et al. (2016)
**Health care utilization and cost**	
Hospitalization	Ackermann et al. (2003, 2008); Nguyen et al. (2007)
Total health care costs	Ackermann et al. (2003, 2008); Nguyen et al. (2007)
Primary care visits and costs	Ackermann et al. (2003, 2008); Nguyen et al. (2007)
Diabetes-related visits	Nguyen et al. (2007)
Specialty care visits and costs	Ackermann et al. (2008)
Inpatient costs	Ackermann et al. (2003, 2008); Nguyen et al. (2007)
**Other**	
Self-rated health status	Batra et al. (2019); Palmer et al. (2016); Steinman et al. (2024); Tomioka et al. (2024)
Number of falls	Batra et al. (2019)
Falls requiring medical treatment	Greenwood-Hickman et al. (2015)
Medical Outcomes Study Short-Form 36	Wallace et al. (1998)
Medical Outcomes Study Short-Form 12	Jones et al. (2024)
Blood pressure	Sin et al. (2005) ^a^
Satisfaction	Jones et al. (2024); Sin et al. (2005)
Arthritis pain intensity	Jones et al. (2024)

a No statistically significant improvement reported.

Five studies reported adverse events, including pain flares (*n* = 3), falls (*n* = 3), and muscle strains (*n* = 1). All reported adverse events were minor and managed with minimal disruption in the class. Subgroup analyses were conducted across studies and included frequency of attendance, dosage levels, completion versus non-completion of EF classes, race, self-rated confidence to use the web, former versus current EF participants, age, delivery site type, year, and exercise frequency.

#### Adoption

Geographically, the most frequent adoption occurred in Washington State, South Florida, and Hawai‘i, with additional adoption in California, West Virginia, Michigan and other rural and urban communities. Broader dissemination was also documented, ranging from regional networks (e.g., 10 YMCA associations in six states, 19 sites in Hawai‘i) to national efforts. The largest dataset drew on EF participation across 41 states and Washington, D.C. The number of sites offering EF varied widely across studies. Some studies focused on a single site or a small number of locations (e.g., 2–3 sites), while others reported adoption across 14–20 sites. Large-scale efforts included 83 sites in one study and 559 sites in another using national secondary data. One multi-site evaluation reported that 17 of 18 invited sites (94%) adopted EF, with a 100% instructor adoption rate at those sites.

Instructor requirements and training were consistently reported. EF instructors were typically required to hold a nationally recognized fitness certification (e.g., American Council on Exercise, American College of Sports Medicine, YMCA) and to complete EF-specific training delivered by EF Master Trainers. Training included pre-training modules, 12-hr to two-day certification programs, supervised practice, and ongoing fidelity monitoring. Instructors came from different professional and community backgrounds, including fitness staff, senior center personnel, aging services staff, extension agents, and volunteers. Adoption rates among instructors varied, with one study reporting that only 55% of trained instructors ultimately led EF classes. Cost-related considerations were reported in some studies. Instructor compensation accounted for a large proportion of program expenses, and agencies sought to reduce costs by recruiting volunteers or low-cost instructors. Several adoption efforts emphasized cultural and linguistic tailoring, such as recruiting bilingual Spanish- and Korean-speaking instructors to serve ethnically diverse participants (see Table S3, Supplementary Appendix 3).

#### Implementation

Overall, most studies followed the standard EF protocol of three sessions per week and 60 min per session. One study reported slightly shorter sessions of 50 min. Program length varied across studies. Several delivered EF for 12 to 16 weeks, others extended to 24 weeks or 12 months, and some offered the program on an ongoing basis within community or organizational settings. A few studies reported shorter interventions (e.g., 6–10 weeks) or multiple cycles. Among the 33 included studies, most delivered EF in person, while 5 studies (15.2%) published after 2020 delivered remotely. Barriers and facilitators to EF implementation were reported across multiple domains and are synthesized in Table 4. Using the CFIR framework, these factors included intervention characteristics (e.g., program adaptability vs delivery costs), outer and inner setting factors (e.g., funding support vs staff capacity), individual-level influences for both participants and instructors (e.g., health benefits vs illness, turnover), and implementation processes (e.g., structured training vs challenges sustaining participation and data collection).

**Table 4:** Barriers and facilitators to EF implementation, organized by consolidated framework for implementation research (CFIR) domains.

CFIR construct	Facilitators	Barriers
Intervention characteristics	Evidence-based and adaptable program; modifications available (e.g., culturally tailored music); variety of exercise resources	Perceived mismatch of intensity (too easy/too hard); need for equipment/internet for tele-EF; delivery costs (licenses, weights, instructor salaries).
Outer setting	Start-up funding; sustained support from partners (AARP, county/state governments); economies of scale (lower cost with more classes); national and community partnerships	Grant dependence or restricted funding; participant fees leading to attrition; seasonal residents (South Florida)
Inner setting	Alignment with organizational mission; leadership support; program champions; centralized data systems; referrals infrastructure; bilingual capacity at some sites; recruitment toolkits and marketing materials	Competing programming and space limitations; limited staff capacity; paperwork burden; lack of substitute staff; difficulty sustaining outreach
Characteristics of individuals—participants	Perceived health and social benefits; group accountability/camaraderie; encouragement from family/physicians; convenient locations/times; free/low-cost offerings.	Illness, pain flares, frailty, depression; low readiness/motivation; belief of “already active”; technology discomfort (tele-EF); transportation/scheduling conflicts; lower completion among some subgroups (e.g., women facing multiple roles).
Characteristics of individuals—instructors	Nationally certified instructors with EF-specific training; mentoring and fidelity monitoring; bilingual instructors (Spanish/Korean); instructor ownership/enthusiasm	Instructor turnover; loss of interest/job changes; few master trainers in some states; limited substitutes
Implementation process	Structured training by EF Master Trainers; ongoing mentoring/fidelity monitoring; in-class tech support for tele-EF; regular follow-ups with participants	Inconsistent participation/dose; difficulty recruiting men; loss of instructors after training; COVID-19 interruptions; challenges collecting evaluation data

*Note*. CFIR = Consolidated Framework for Implementation Research; AARP = American Association of Retired Persons; COVID-19 = 2019 Novel Coronavirus Acute Respiratory Disease.

Fidelity of EF delivery was explicitly reported in 10 of the 33 included studies (30.3%). Reported approaches included the use of standardized fidelity monitoring tools, random class observations, and supervision of newly trained instructors by EF Master Trainers. Several studies conducted structured fidelity assessments at regular intervals (e.g., baseline, 1 month, and every 4 months), with ratings showing that instructors generally improved over time. Common fidelity concerns included shortened class duration (<60 min), omission of safety reminders, limited balance exercises, or improper sequencing of warm-up/cool-down activities.

#### Maintenance

Maintenance was described at both the program level (i.e., sustained delivery within a given setting and cost) and the participant level (i.e., sustained health outcomes over time). At the program level, 16 studies described continued EF delivery. Examples included integration into usual services for Area Agencies on Aging, sustained partnerships with health care systems (e.g., Group Health Cooperative reimbursement of per-visit costs), and ongoing classes in community settings such as senior centers and YMCAs. Multi-site initiatives documented broader continuation, including 70 EF classes across 56 sites after a 3-year funding period, while some programs transitioned to tele-EF during the COVID-19 pandemic. Discontinuation was also reported, typically due to a lack of instructor support or funding. Reporting on the cost of maintenance was rare. One study estimated that the total cost of operating EF across six sites for one year was $180,476 in 2004, serving 132 participants. Recurrent expenses, particularly instructor training, accounted for the majority of costs. Another study reported that average monthly costs per class declined from $1,713 in the first year to $873 in the second year. At the participant level, follow-up assessments of health outcomes ranged from short-term (2 months) to extended evaluations up to 6 years. The top three common follow-up time points were at 4 months, 6 months, or 12 months. Details were presented in Table S4, Supplementary Appendix 3.

### Study limitations as noted by authors

Most of the authors described study limitations. Among the most often mentioned were inclusion of participants from selected settings, convenience samples, and self-selected participants, which limited the generalizability and potentially future broad applicability of the findings. Pilot studies were not powered to detect meaningful change in outcome measures. The majority of outcomes were assessed immediately at post-intervention without long-term follow-up; therefore, long-term impact was not determined. Studies that used secondary data noted that variables were restricted to those collected under the original grant requirements and that missing data were of concern. Methodological issues around using a single-group design with the lack of a comparison group or randomization meant the authors could not solely attribute the changes in health outcomes over time to EF participation. Studies conducted during and post the COVID-19 pandemic faced methodological and logistical challenges. Although adjustments for differences in age, gender, comorbidity, and baseline in major health care cost and utilization measures were made, the authors were unable to adjust for possible differences in other lifestyle factors that might have made an impact on health care use. Many sites offering EF had limited capacity for outreach. A challenge related to conducting exercise feasibility studies is the tendency for people who already exercise, or are interested in exercise, to be the first respondents to recruitment notices. Key to future implementation is the need for strategies to reach and recruit older adults who may not place a high value on exercise to participate.

## Discussion

This scoping review systematically mapped and synthesized the literature on EF, using the RE-AIM framework, to identify gaps and opportunities to strengthen EF’s dissemination. The results indicate that EF reached racially and ethnically diverse older adults; however, there was a need for greater inclusion of people living with chronic conditions such as dementia or functional limitations. EF was associated with improvements in physical function despite substantial heterogeneity in the outcome measures used. There was underreporting of the RE-AIM domains, participant demographics and clinical characteristics, and fidelity. Site and instructor adoption varied across the United States and implementation barriers and facilitators were well-documented. Maintenance was largely underreported in terms of site sustainability, primarily because delivery was linked to funding.

Across more than three decades of evaluation, EF has evolved from a single community exercise program into a widely disseminated, instructor-led model operating in clinical–community ecosystems. The literature mapped in this scoping review showed consistent improvements in frequently measured physical-function outcomes (e.g., chair rise, arm curl, up-and-go, balance) and a smaller but growing body of work addressing psychosocial outcomes (e.g., loneliness, depressive symptoms, sleep) and health-care utilization and costs. Although EF is an evidence-based program, outcome heterogeneity remains substantial. Investigators used a wide array of measures across physical, psychosocial, and utilization domains, assessed at varying follow-up intervals. While this variability may reflect efforts to explore diverse aspects of EF’s impact, it also complicates cross-study synthesis, potentially masks dose–response relationships, and limits benchmarking of effect sizes across settings. Although not a requirement of publications about EF, reporting across RE-AIM domains was somewhat uneven. Maintenance, both at the participant level (continuation and retention) and the organizational level (program continuation after start-up support), was less frequently and less consistently documented than reach or effectiveness.

Although roughly half the studies noted continued delivery at the site level, few followed programs long enough to characterize sustainment or costs beyond the first year, and only a small subset quantified resources required to maintain classes. Another notable issue was recruitment often relied on self-selection through community channels that preferentially attract individuals already inclined toward group exercise. Demographic and clinical subgroup reporting was inconsistent; stratified analyses by race/ethnicity, language, socioeconomic status, or multimorbidity were limited; and cognitive impairment was frequently an exclusion criterion. These features constrain generalizability and risk widening participation gaps. Cumulatively, these limitations reflect a field that has matured in dissemination but still needs more harmonized measurement, the application of transparent and reproducible designs, and more deliberate attention to equity and the costs for sustaining EF to support confident policy and financing decisions.

### Implications

Further implementation efforts have an opportunity to strategize around the elements that appear to drive observed benefits and widen participation. Instructors typically hold national certifications and complete EF-specific training, which can be leveraged to support ongoing supervision and planning. As previously mentioned, recruitment skews toward those already inclined to exercise and participation is inconsistent; partnerships with primary care and aging services, along with bilingual delivery that sites have already implemented, are credible paths to a more representative reach and steadier dose. Only a handful of studies used virtual formats after 2020 (Gell et al., 2023, 2024; Steinman et al., 2024; Tomioka et al., 2024), and although some technology discomfort was documented, sites that provided in-class troubleshooting reported it as a practical facilitator; hybrid or remote offerings could be offered to help avoid attrition and preserve the group format that defines EF. Future studies are needed to quantify costs associated with virtual delivery and sustainability.

Given the documented effectiveness of EF in the United States and its established presence in the aging network (Sound Generations, 2025), reviews such as this may support greater adoption in clinical settings and guide health policy. Examples from existing healthcare and community partnerships illustrate the feasibility of broader EF dissemination. EF has been integrated into large regional health systems through Medicare Advantage–supported participation models and is also widely delivered through national community infrastructure, such as YMCA networks across over 40 states. EF represents a lower-cost intervention that can bridge clinical and community practice to give older adults additional opportunities for healthful activities to manage chronic conditions and curb their progression. Awareness-raising efforts can inform clinicians about the benefits of EF and how to create dedicated referral processes to connect older adults to community sites offering the program. To support these efforts, policies are needed to create and promote billable codes for EF referrals, incentivizing clinicians for referring patients to workshops, and establishing reimbursements to community organizations that serve healthcare patients. Future studies are needed to objectively assess the association of EF participation with healthcare utilization and financial return on investment for insurers, clinical sites, and community organizations. In addition, many included studies were conducted during earlier phases of national dissemination, when implementation infrastructure, costs, and delivery processes have differed from current practice. As EF has matured and expanded, program operations and resource allocation have evolved. Continued contemporary research is needed to capture ongoing adaptations and implementation within changing healthcare and community contexts.

Despite growing recognition of the importance of physical activity for older adults, people living with dementia remain significantly less active than their cognitively healthy older adults (Muurling et al., 2023). In this scoping review, people living with dementia were often excluded from EF participation, either implicitly through lack of characterization or explicitly as an exclusion criterion. Importantly, these exclusions were related to study-specific research protocols and do not necessarily reflect routine EF class participation practices. This represents a missed opportunity to extend the program’s reach and address a population with considerable need and potential benefit. Dementia was rarely characterized among participants and was explicitly an exclusion criterion in some studies, which narrows the program’s broader public-health reach. EF’s supervised, standardized format and trained instructor workforce suggest strong potential for adaptation to better support participants living with dementia. EnhanceFitness instructors are guided to use evidence-informed strategies, including slower pacing, enhanced verbal and visual cueing with repetition, simplified instructions, and predictable sequencing to reduce cognitive load. Environmental adaptations (e.g., minimizing background noise, maintaining consistent room setup), increased safety monitoring, and success-oriented movement modifications further support engagement. Involvement of caregivers or staff and attention to non-verbal cues of fatigue or confusion can additionally enhance safe and meaningful participation. Priorities for evaluation include inclusion of people living with dementia with appropriate safety and customized measures, reporting of cognitive status, and assessment of engagement and function in both in-person and hybrid formats.

The Finnish Geriatric Intervention Study demonstrated that a 2-year multidomain intervention combining diet, physical activity, cognitive training, and vascular risk monitoring can slow cognitive decline in at-risk older adults (Kivipelto et al., 2013). The U.S. POINTER randomized clinical trial reported that both a structured and a lower-intensity self-guided multidomain lifestyle program improved cognition over 2 years, with greater gains in the structured arm (Baker et al., 2025). These trials support integration of structured physical activity within broader, community-deployable packages that target multiple risk factors for brain health, and they justify testing EF as the physical-activity component of such interventions, including adaptations for people living with dementia.

### Limitations

Our scoping review had several limitations. First, no quality appraisal of the included studies was conducted, which is characteristic of scoping reviews. Second, studies included in this scoping review had wide variability in the measures used and outcomes reported. Third, we relied on published literature and therefore may have excluded relevant but unpublished program evaluations, internal organizational reports, or grey literature. Fourth, given the long-standing research history of EF, some included studies of shared authorship and, in certain cases, overlapping program samples. Although we assessed for duplicate reporting and retained only distinct analyses, residual overlap may remain and could influence the synthesis. Fifth, we did not systematically compare pre- and post-COVID-19 implementation environments. The pandemic prompted significant shifts in EF delivery, including the rapid transition to remote formats, which may have meaningfully affected reach, fidelity, and participant outcomes. Future reviews with sufficient post-pandemic studies may be better positioned to systematically examine these differences.

## Conclusion

This scoping review systematically mapped and synthesized 33 data-based publications on EF, using the RE-AIM framework. Unique contributions of the review include multi-method evidence synthesis, dual theoretical integration, and a forward-looking research and equity agenda. Our findings provide greater context for the implementation and dissemination of EF in terms of the RE-AIM framework. Continued access to EF for future generations of older adults requires targeted efforts examining expanded reach, widening inclusivity approaches (e.g. adaptations for people living with dementia and their care partners), continued evaluation of implementation options (e.g. remote delivery, modified duration), and a focus on supports needed for long-term maintenance and engagement.

## Supplementary Material

gnag102_Supplementary_Data

## Data Availability

The data synthesized in this scoping review are derived from previously published studies, which are available through academic databases. All data extracted and generated for this review are included within the article and its Supplementary Material. Specifically, the comprehensive extraction data are available in Supplementary Appendix 3, and the search strategies are provided in Supplementary Appendix 2. This review was preregistered with the Open Science Framework (https://doi.org/10.17605/OSF.IO/PCFDX).
