# Supplementary material for: Implementation of Enhance®Fitness: a scoping review using the RE-AIM framework
Source: Gerontologist. 2026 May 9;66(7):gnag102. doi: 10.1093/geront/gnag102 (PMC13310477; doi:10.1093/geront/gnag102)
Supplement: gnag102_Supplementary_Data [file gnag102_supplementary_data.pdf]

Title: Implementation of Enhance®Fitness: A scoping review using the RE-AIM framework

Authors: Wenting Peng; Sarah McKiddy; Michiyo Tomioka; Dina L. Maruca; Nancy M. Gell; Matthew Lee Smith; Caitlin Maloy; Basia Belza

Appendix (Supplemental Material for Online Publication)

Appendix 1

(PRISMA-ScR) Checklist

Appendix 2

Searching Strategy

Appendix 3

Table S1. Study Characteristics
Table S2. Reach
Table S3. Adoption
Table S4. Maintenance

Appendix 1 (PRISMA-ScR) Checklist

Preferred Reporting Items for Systematic reviews and Meta-Analyses extension for Scoping Reviews (PRISMA-ScR) Checklist

SECTION	ITEM	PRISMA-ScR CHECKLIST ITEM	REPORTED ON PAGE #
**TITLE**			
Title	1	Identify the report as a scoping review.	#1
**ABSTRACT**			
Structured summary	2	Provide a structured summary that includes (as applicable): background, objectives, eligibility criteria, sources of evidence, charting methods, results, and conclusions that relate to the review questions and objectives.	#2-3
**INTRODUCTION**			
Rationale	3	Describe the rationale for the review in the context of what is already known. Explain why the review questions/objectives lend themselves to a scoping review approach.	#4-5
Objectives	4	Provide an explicit statement of the questions and objectives being addressed with reference to their key elements (e.g., population or participants, concepts, and context) or other relevant key elements used to conceptualize the review questions and/or objectives.	#5
**METHODS**			
Protocol and registration	5	Indicate whether a review protocol exists; state if and where it can be accessed (e.g., a Web address); and if available, provide registration information, including the registration number.	#6
Eligibility criteria	6	Specify characteristics of the sources of evidence used as eligibility criteria (e.g., years considered, language, and publication status), and provide a rationale.	#6
Information sources*	7	Describe all information sources in the search (e.g., databases with dates of coverage and contact with authors to identify additional sources), as well as the date the most recent search was executed.	#6
Search	8	Present the full electronic search strategy for at least 1 database, including any limits used, such that it could be repeated.	#6
Selection of sources of evidence†	9	State the process for selecting sources of evidence (i.e., screening and eligibility) included in the scoping review.	#7
Data charting process‡	10	Describe the methods of charting data from the included sources of evidence (e.g., calibrated forms or forms that have been tested by the team before their use, and whether data charting was done independently or in duplicate) and any processes for obtaining and confirming data from investigators.	#7
Data items	11	List and define all variables for which data were sought and any assumptions and simplifications made.	N/A
Critical appraisal of individual sources of evidence§	12	If done, provide a rationale for conducting a critical appraisal of included sources of evidence; describe	N/A

SECTION	ITEM	PRISMA-ScR CHECKLIST ITEM	REPORTED ON PAGE #
		the methods used and how this information was used in any data synthesis (if appropriate).	
Synthesis of results	13	Describe the methods of handling and summarizing the data that were charted.	#8
**RESULTS**			
Selection of sources of evidence	14	Give numbers of sources of evidence screened, assessed for eligibility, and included in the review, with reasons for exclusions at each stage, ideally using a flow diagram.	#8
Characteristics of sources of evidence	15	For each source of evidence, present characteristics for which data were charted and provide the citations.	#8
Critical appraisal within sources of evidence	16	If done, present data on critical appraisal of included sources of evidence (see item 12).	N/A
Results of individual sources of evidence	17	For each included source of evidence, present the relevant data that were charted that relate to the review questions and objectives.	#12
Synthesis of results	18	Summarize and/or present the charting results as they relate to the review questions and objectives.	#15
**DISCUSSION**			
Summary of evidence	19	Summarize the main results (including an overview of concepts, themes, and types of evidence available), link to the review questions and objectives, and consider the relevance to key groups.	#15
Limitations	20	Discuss the limitations of the scoping review process.	#18
Conclusions	21	Provide a general interpretation of the results with respect to the review questions and objectives, as well as potential implications and/or next steps.	#18
**FUNDING**			
Funding	22	Describe sources of funding for the included sources of evidence, as well as sources of funding for the scoping review. Describe the role of the funders of the scoping review.	#1

JBI = Joanna Briggs Institute; PRISMA-ScR = Preferred Reporting Items for Systematic reviews and Meta-Analyses extension for Scoping Reviews.

* Where *sources of evidence* (see second footnote) are compiled from, such as bibliographic databases, social media platforms, and Web sites.

† A more inclusive/heterogeneous term used to account for the different types of evidence or data sources (e.g., quantitative and/or qualitative research, expert opinion, and policy documents) that may be eligible in a scoping review as opposed to only studies. This is not to be confused with *information sources* (see first footnote).

‡ The frameworks by Arksey and O'Malley (6) and Levac and colleagues (7) and the JBI guidance (4, 5) refer to the process of data extraction in a scoping review as data charting.

§ The process of systematically examining research evidence to assess its validity, results, and relevance before using it to inform a decision. This term is used for items 12 and 19 instead of "risk of bias" (which is more applicable to systematic reviews of interventions) to include and acknowledge the various sources of evidence that may be used in a scoping review (e.g., quantitative and/or qualitative research, expert opinion, and policy document).

From: Tricco AC, Lillie E, Zarin W, O'Brien KK, Colquhoun H, Levac D, et al. PRISMA Extension for Scoping Reviews (PRISMAScR): Checklist and Explanation. Ann Intern Med. 2018;169:467–473. doi: 10.7326/M18-0850.

Appendix 2 Searching Strategy

PubMed

("Lifetime Fitness Program" OR "EnhanceFitness" OR “Enhance®Fitness” OR “Enhanced Fitness” OR “Enhance_Fitness” OR “Enhance Fitness” OR "Tele-EF"[tiab] OR ("EF"[tiab] AND "Physical Fitness"[Mesh])) AND (aged[Filter] OR "Aged"[Mesh] OR aged OR aging OR elderly OR old OR "older adult") AND (1980:2025[pdat]) NOT (allchild[Filter] OR "Child"[Mesh] OR "Adolescent"[Mesh] OR "Pediatrics"[Mesh] OR child OR children OR adolesc* OR teen OR teenager OR youth OR pediatr*) NOT ("Review" [Publication Type] OR "Editorial" [Publication Type] OR "Clinical Conference" [Publication Type] OR editorial OR conference)

CINAHL

(("Lifetime Fitness Program" OR "EnhanceFitness" OR “Enhance®Fitness” OR “Enhanced Fitness” OR “Enhance_Fitness” OR “Enhance Fitness” OR TI "Tele-EF" OR AB "Tele-EF" OR ((TI "EF" OR AB "EF") AND (MH "Physical Fitness+"))) AND (MH "Aged+" OR aged OR aging OR elderly OR old OR “older adult”) AND (PY 1980-2025) NOT (MH "Child+" OR MH "Adolescence+" OR MH "Pediatrics+" OR child OR children OR adolesc* OR teen OR teenager OR youth OR pediatr*) NOT (MH "Literature Review+" OR MH "Clinical Conferences" OR editorial OR conference))

Embase

('lifetime fitness program':ti,ab OR 'enhancefitness':ti,ab OR 'enhanced fitness':ti,ab OR 'enhance_fitness':ti,ab OR 'enhance fitness':ti,ab OR 'tele-ef':ti,ab OR ('ef' AND ('fitness'/exp OR 'physical fitness'))) AND ('aged'/exp OR 'aging'/exp OR aged OR aging OR elderly OR old OR 'older adult') AND ([1980-2025]/py) AND ([embase]/lim) NOT ('juvenile'/exp OR 'pediatrics'/exp OR children OR adolesc* OR teen OR teenager OR youth OR pediatr*) NOT ('conference paper'/exp OR 'editorial'/exp OR 'conference abstract'/it OR 'conference paper'/it OR 'editorial'/it OR 'review'/it OR editorial OR conference)

Web of Science

((TI=("Lifetime Fitness Program" OR "EnhanceFitness" OR “Enhance®Fitness” OR “Enhanced Fitness” OR “Enhance_Fitness” OR “Enhance Fitness” OR "Tele-EF" OR ("EF" AND "Physical Fitness”))) OR (AB=("Lifetime Fitness Program" OR "EnhanceFitness" OR “Enhance®Fitness” OR “Enhanced Fitness” OR “Enhance_Fitness” OR “Enhance Fitness” OR "Tele-EF" OR ("EF" AND "Physical Fitness”)))) AND (ALL=(aged OR aging OR elderly OR old OR "older adult")) AND (PY=(1980-2025)) NOT (ALL=(children OR adolesc* OR teen OR teenager OR youth OR pediatr*)) NOT (ALL=(editorial OR conference))

PsycINFO

("Lifetime Fitness Program" OR "EnhanceFitness" OR “Enhance®Fitness” OR “Enhanced Fitness” OR “Enhance_Fitness” OR “Enhance Fitness” OR TI "Tele-EF" OR AB "Tele-EF" OR ((TI "EF" OR AB "EF") AND (DE "Physical Fitness"))) AND (DE "Aged (Attitudes Toward)" OR DE "Aging" OR DE "Older Adulthood" OR aged OR aging OR elderly OR old OR “older adult”) AND (PY 1980-2025) NOT (DE

"Pediatrics" OR child OR children OR adolesc* OR teen OR teenager OR youth OR pediatr*) NOT (editorial OR conference)

Cochrane

("Lifetime Fitness Program" OR "EnhanceFitness" OR “Enhance®Fitness” OR “Enhanced Fitness” OR “Enhance_Fitness” OR “Enhance Fitness” OR Tele-EF OR (EF AND "Physical Fitness”)) AND (aged OR aging OR elderly OR old OR "older adult") NOT (children OR adolesc* OR teen OR teenager OR youth OR pediatr*)

Appendix 3

Table S1. Study Characteristics
Table S2. Reach
Table S3. Adoption
Table S4. Maintenance

Table S1. Study Characteristics

Study	Title	Purpose	Study Design	Sample Size	Setting
Ackermann (2003)	Community Exercise Program Use and Changes in Healthcare Costs for Older Adults	This study was conducted to determine if changes in healthcare costs or utilization for Medicare-eligible enrollees of a large health maintenance organization (HMO) are related to their choice to participate in a community exercise program that is offered as a health benefit. (p. 233)	Other: Retrospective, matched cohort study	4456<br>LFP: n = 1114<br>control: n = 3342	Clinical setting
Ackermann (2008)	Healthcare Cost Differences with Participation in a Community-Based Group Physical Activity Benefit for Medicare Managed Care Health Plan Members	This new study was conducted to extend our prior analyses by examining the association between EF participation and healthcare utilization and costs beyond 1 year and incorporating information about the use of other preventive services to reduce the potential for residual confounding and selection bias. (p., 1460)	Retrospective (case-control, historical prospective); Prospective (cohort, longitudinal); Quasi-experimental	3650	Clinical setting; Community setting
Agmon (2015)	The Effects of EnhanceFitness (EF) Training on Dual-Task Walking in Older Adults	To explore whether an evidence-based group exercise program (EnhanceFitness [EF]) that has demonstrated improvement in balance, muscle strength, flexibility, and endurance (NP130)	Quasi-experimental	28	Community setting
Batra (2016)	Evaluating the Factors Associated With the Completion of a Community-Based Group Exercise Program Among Older Women	The purpose of the current study was to identify factors associated with the completion of EF program (p. 649)	Prospective (cohort, longitudinal)	3829	Community setting
Batra (2019)	Determining the Long-Term Effectiveness of a Group-Based Physical Activity Program	The purpose of this study is to assess the extended effectiveness of EF participation from baseline to 12 months. The lack of literature on long-term (4-, 8-, and 12 months) impacts of EF participation makes this study valuable. (p. 402)	Prospective (cohort, longitudinal); Quasi-experimental	1295	Clinical setting; Community setting; Other: skilled nursing facilities, assisted living facilities, or senior housing
Belza (2015)	Adoption of evidence-based health promotion programs: perspectives of early adopters of	what are the facilitators and barriers among early adopters of EF in Y-affiliated sites from the perspective of program staff...we provide practice, research, and policy	Qualitative	15	Community setting

	Enhance®Fitness in YMCA-affiliated sites	recommendations that may help inform the adoption of other evidence-based programs in community settings. p 2			
Biedenweg (2014)	Understanding Older Adults' Motivators and Barriers to Participating in Organized Programs Supporting Exercise Behaviors	In order to better understand why older persons are not participating in physical activity-oriented programs designed specifically for them, we undertook a qualitative study involving older adults who had elected or declined to participate in such a program. Our purpose was to explore motivators and barriers to program participation among these individuals. We also sought to understand how program marketing strategies had influenced their decision to join a particular program and what additional or alternative marketing strategies might be recommended (p. 2)	Qualitative	39<br>program joiners: n = 19<br>decliners: n = 20	Community setting
Chiang (2008)	"It Is Our Exercise Family": Experiences of Ethnic Older Adults in a Group-Based Exercise Program	We examined how physical environment, social environment, and individual biology and behavior influenced adherence to exercise among ethnic older adults participating in EF. Our goal is to use the information to generate effective strategies to promote adherence to exercise programs among ethnic older adults. The information will also be useful for future EF program evaluation and development. (p. 3)	Qualitative	52	Community setting
Fishleder (2019)	Predictors of Improvement in Physical Function in Older Adults in an Evidence-Based Physical Activity Program (EnhanceFitness)	The purpose of this article was to examine changes in, and predictors of, participant physical function from baseline through 2 program cycles of EF as measured by 3 physical function tests: arm curls, chair stands, and 8-foot up-and-go. We examined the association between changes in physical function with EF attendance and participant-level characteristics. (p. 231)	Prospective (cohort, longitudinal)	7483	Community setting

Gell (2021)	Technology Support Challenges and Recommendations for Adapting an Evidence-Based Exercise Program for Remote Delivery to Older Adults: Exploratory Mixed Methods Study	to (1) examine the needs of older adults previously enrolled in community-based exercise for transition to tele-exercise, (2) identify barriers to and facilitators of tele-exercise uptake and continued participation, and (3) describe technology support challenges and successes encountered by older adults starting tele-exercise (p.2)	Mixed methods	44	Home
Gell (2023)	Exercise Intensity Among Older Adults Participating From Home in Remotely Delivered EnhanceFitness	Therefore, the primary purpose of this study was to examine exercise intensity through heart rate (HR) monitoring and ratings of perceived exertion (RPE) during remotely delivered EnhanceFitness (Tele-EF). The secondary purpose was to examine participant health and demographic characteristics with duration of moderate-intensity PA during Tele-EF. Of particular interest was whether participants with more impaired physical function at baseline would have fewer minutes at moderate intensity.	Cross-sectional	55	Home
Gell (2024)	Remotely delivered exercise to older rural cancer survivors: a randomized controlled pilot trial	We sought to determine the feasibility of tele-EF for older, rural cancer survivors and to explore the preliminary effects of tele-exercise on physical function, physical activity, and patient-reported outcomes. (p. 597)	Prospective (cohort, longitudinal); Randomized controlled trial	39	Home
Gillette (2015)	A Pilot Study of Determinants of Ongoing Participation in EnhanceFitness, a Community-Based Group Exercise Program for Older Adults	In this study we address these gaps in the literature by identifying motivators and barriers for participation in EF and by determining if identified motivators and barriers are predictive to dropping out.	Cross-sectional	241	Community setting
Greenwood-Hickman (2015)	Participation in Older Adult Physical Activity Programs and Risk for Falls Requiring Medical Care, Washington State, 2005–2011	Our objective was to examine the relationship between participation in EF or SS and risk for a fall requiring medical treatment (termed “medical fall” hereafter), in a sample of GHC Medicare plan enrollees.	Prospective (cohort, longitudinal)	70,582	Community setting

Jones (2024)	Effectiveness and Implementation of an EnhanceFitness Physical Activity Intervention Specifically in Adults With Arthritis: A RE-AIM Evaluation	The primary aim of this study was to evaluate the effectiveness of 12 weeks of EF in sedentary/low-active adults with self-reported physician-diagnosed arthritis, across the adult population age spectrum, under real-world conditions, in both urban and rural WV communities. Our secondary aim was to evaluate the implementation of EF. We used the RE-AIM Framework to evaluate EF's: (1) reach (enrollment); (2) effectiveness (outcomes); (3) adoption (proportion of sites/instructors that delivered EF); (4) implementation (attendance, fidelity, adverse events, and satisfaction); and (5) maintenance (EF continuation). (p. 2)	Other: Nonrandomized controlled trial	323	Community setting
Kohn (2015)	Participant Variation by Delivery Site Type in an Evidence-Based Physical Activity Program	To describe EF participant characteristics and physical function test results by delivery site type (p. 3)	Prospective (cohort, longitudinal)	19,964	Community setting
Kohn (2016)	Beyond Strength: Participant Perspectives on the Benefits of an Older Adult Exercise Program	The purpose of this article is to describe the expected and experienced benefits among older adult participants in EF, and the implications for program dissemination, particularly program reach, implementation, and maintenance.	Qualitative	20	Community setting
Mays (2021)	The Leveraging Exercise to Age in Place (LEAP) Study: Engaging Older Adults in Community-Based Exercise Classes to Impact Loneliness and Social Isolation	We aimed to demonstrate the impact of exercise based EBPs on loneliness and social isolation as these programs would offer a scalable intervention with multifaceted benefits for older adults. Our initial primary hypothesis was that the DSSI would improve by 4 points following EBP participation and be maintained at 6-month. Our secondary hypotheses were that EBP participation would decrease falls risk and health care utilization and these analyses will be conducted in the future (p. 2)	Quasi-experimental	382	Clinical setting; Other: self-referred from the surrounding community

Nguyen (2007)	Impact of a managed-Medicare physical activity benefit on health care utilization and costs in older adults with diabetes	The purpose of this study was to determine the effects of a managed-Medicare physical activity benefit on both outpatient and inpatient service utilization and costs among older adults with diabetes.	Prospective (cohort, longitudinal)	527<br>EF participants: n = 163<br>control subjects: n = 364	Community setting
Page (2014)	Implementation Cost Analysis of a Community-Based Exercise Program for Seniors in South Florida	The purpose of this study was to measure the costs associated with EnhanceFitness implementation in South Florida. The current study had two objectives:<br>1. To provide data on the total yearly cost of implementing and delivering EnhanceFitness classes<br>2. To compute the per month cost for each class offered. (p. 586)	Retrospective (case-control, historical prospective)	7 agencies (1969 participants)	Community setting
Palmer (2016)	Implementation of an Evidence-Based Exercise Program for Older Adults in South Florida	Therefore, we investigated the ability of HARC to implement and sustain the EF program in South Florida over a three-year period. We examine if HARC was able to retain program fidelity, recommended dosage and exposure to the program, and comparable gains in functional fitness.	Quasi-experimental	4,490	Community setting
Patel (2022)	Remotely Delivered Exercise to Rural Older Adults With Knee Osteoarthritis: A Pilot Study	to evaluate the feasibility and acceptability of remotely delivered EF (tele-EF) among rural older adults with knee OA. (p.736)	Quasi-experimental	15	Home;<br>Other: Delivered via Zoom videoconferencing
Petrescu-Prahova (2016)	Implementation and Maintenance of a Community-Based Older Adult Physical Activity Program	To examine the implementation and maintenance of Enhance®Fitness (EF) at early-adopter YMCA-affiliated sites (p. 1-2)	Qualitative	32	Community setting

Petrescu-Prahova (2022)	Clinical-community linkages as a strategy for increasing evidence-based program reach: Results of the PT-REFER randomized controlled trial with older adults and YMCA associations	1. Purpose was stated on p. 3: Our purpose was to improve Association linkages to PT organizations and providers to increase older adult PA via EF enrollment. (p. 3)<br>2. We hypothesized that implementing the intervention would lead to increased older adult enrollment in EF compared to usual delivery of EF. Specifically, we hypothesized that Associations receiving the intervention would increase their time on, and their effectiveness with, outreach to PTs, and these PTs would then refer their older-adult clients to EF. (p. 2)<br>3. We report here the outcomes of the Physical Therapists–Recommending Enhance®Fitness to Expand Reach (PT-REFER) trial, testing a capacity building intervention with Young Men's Christian Association (YMCA) Associations (hereafter referred to as Associations) to form CCLs with physical-therapy clinics, thereby increasing PT referrals to Enhance®Fitness (EF). (p. 2)	Randomized controlled trial	20	Clinical setting; Community setting; Other: Clinical-community linkage
Rosenberg (2014)	Characteristics of Older Adult Physical Activity Program Users	...To examine recent use patterns of the programs and to identify the characteristics of older adults who use the programs, compared with nonusers, to obtain insight into how characteristics efforts might be best targeted to improve program uptake. (p. 2 or 246)	Cross-sectional	37,492	Community setting
Sin (2005)	Evaluation of a Community-Based Exercise Program for Elderly Korean Immigrants	To evaluate feasibility and effectiveness of a modified exercise program for elderly Korean immigrants (EKIs)	Quasi-experimental; Mixed methods	13	Community setting

Smith (2014)	National Reach and Dissemination of EnhanceFitness	We: (1) examine the dissemination of EF by grantee state, delivery site type, and participant characteristics during the AoA-sponsored EBDDP initiative; (2) describe strategies and lessons learned pertaining to participant and delivery site recruitment and retention using state-specific case examples; and (3) discuss policy and programmatic implications for the delivery of EF at the national, state, and local levels. (p.151)	Retrospective (case-control, historical prospective); Mixed methods	5719	Community setting; Other: health care organization, Faith-based Organization, Recreational Organization, Senior Center, Multi-purpose Social Services Organization, Other Community Center, Parks Department Facility, Residential Facility, Other
Steinman (2024)	Remote Evidence-Based Programs for Health Promotion to Support Older Adults During the COVID-19 Pandemic and Beyond: Mixed Methods Outcome Evaluation	The study aim/objective was to conduct a national mixed methods outcome evaluation of remotely delivered evidence-based programs (EBPs) that transitioned from in-person to remote formats due to the COVID-19 pandemic, focusing on older adults. The evaluation assessed changes in multiple health and well-being outcomes including self-rated health, fatigue or energy, exercise, pain, sleep, depression, anxiety, loneliness, social isolation, and program-specific outcomes (p. 4).	Qualitative; Prospective (cohort, longitudinal); Quasi-experimental; Mixed methods	12	Community setting
Sugihara (2011)	Cost–Benefit Estimates of an Elderly Exercise Program on Kaua‘i	This paper describes Kaua‘i’s EF program, its cost and fidelity to the original program, which are critical to extrapolating results from the original study. (p. 116)	Cross-sectional; Retrospective (case-control, historical prospective)	27	Community setting
Tomioka (2012)	Replicating the EnhanceFitness Physical Activity Program in Hawai`i’s Multicultural Population, 2007-2010	The purpose of this article is to describe how Kaua`i County selected, adapted, implemented, and evaluated EnhanceFitness to increase availability of physical activity programs and physical performance among older adults. (p. 2)	Prospective (cohort, longitudinal)	223	Community setting

Tomioka (2019)	Twelve-Month Retention in and Impact of Enhance® Fitness on Older Adults in Hawai'i	This paper fills a gap by presenting findings from a longer-term (12-month) analysis of Enhance®Fitness physical performance data from a sample that includes large proportions of Asians and NHPI elders and adding to the research on program drop out (p. 2)	Prospective (cohort, longitudinal)	427	Community setting
Tomioka (2024)	The Impact of the COVID-19 Pandemic on Older Adults Who Participate in Group Physical Exercise Program	This study explored how the pandemic impacted older-adult participants who were part of the Enhance®Fitness in Hawai'i, examined the longer-term feasibility of offering physical exercise programs remotely, and identified important factors related to designing accessible health promotion services and programs with cultural considerations for the future (p. 180-181).	Cross-sectional	291	Community setting; Home
Wallace (1998)	Implementation and Effectiveness of a Community-based Health Promotion Program for Older Adults	The purpose of this pilot study was to evaluate the feasibility and efficacy of delivering an integrated disability-prevention intervention at a neighborhood senior center (p. 302)<br>The long-term goal of this research is to develop and test a cost-effective and practical community-based disability-prevention intervention for older adults (p. 302)	Randomized controlled trial	100<br>intervention group: n = 53<br>control group: n = 47	Community setting

Table S2. Reach

Study	Target population	Inclusion criteria	Exclusion criteria	Recruitment Source	Representativeness of participants	Equity considerations
Ackermann (2003)	Medicare-eligible adults who choose to participate in a community-based exercise program	Two matched cohorts were sampled from patients aged 65 years who were continuously enrolled in GHC between October 1997 and December 2000. The exposure cohort included all eligible GHC enrollees who participated in the exercise program at least once. A frequency matching procedure based on age and gender was used to select three enrollees, who had never participated in the exercise program, to serve as controls for each program participant. (p. 233)	Not reported	Existing client database	Age: Control 74.9 and Intervention 75 years (SD Not reported)<br>Female %: 75.3% in control and 75.3% in participants	Not reported
Ackermann (2008)	Medicare enrollees who were aged 65 and older, were enrolled in GHC between October 1, 1997, and December 31, 2004, and elected to participate in EF at least once were sampled (p.1460)	- Medicare enrollees aged 65 and older<br>- Enrolled in Group Health Cooperative of Puget Sound (GHC), a large health maintenance organization<br>- Enrolled between October 1, 1997, and December 31, 2004<br>- Elected to participate in the EF program at least once (for EF users)<br>- For controls, members who met the age and enrollment criteria but did not attend any EF visits were selected and matched by age and sex	- Baseline long-term care utilization<br>- Missing RxRisk comorbidity indicator data<br>- Disenrollment from the health plan<br>- Missing total cost data during the first year after the index date	Existing client database;<br>Other: Members for Group Health	Age: 75.4 and 75.7<br>Female %: 77 and 75.5	Not reported
Agmon (2015)	Community-dwelling older adults, who had not started any new exercise routine for at	(a) age 60 or older, (b) able to walk 10 meters independently, (c) new to EF, and (d) could speak,	(a) any active and untreated symptomatic illness that might limit the participant's ability to	Not reported	Age: 74.5 (7.9)<br>Female %: 89.3	Not reported

	least 2 months prior to the study	understand, and read English.	adhere to the program;<br>(b) presence of neurologic or mus-culoskeletal diagnosis, such as cerebral vascular accident (CVA), Parkinson's disease, Alzheimer's disease, or multiple sclerosis;<br>(c) severe orthopedic restrictions such as acute back pain or a total hip replacement within 3 months prior to the study;<br>(d) significant hearing or vision loss.			
Batra (2016)	Community-dwelling women, aged 60 years and older, who participated in EF between 10/1/08 & 12/31/12 in Broward, Miami-Dade, and Monroe counties in South Florida.	Community-dwelling older adults (60 years or older) who were able to cognitively follow instructions and did not have any medical condition that prohibited exercise such as end stage renal or heart disease. Note that the current manuscript only focused on women.	Not reported	Flyers; Posters; Word of mouth	Age: Mean (SD) = 75.7 (8.6), 60-69 yrs=26.9%, 70-79 yrs=41.6%, GTE 80 yrs=31.5%<br>Female %: 100%<br>Black/African American: 681 (17.8)<br>Hispanic/Latino: 961 (25.1)	Not reported
Batra (2019)	Older adults aged 60 years or older who were cognitively able to follow instructions	1. Aged 60 years or older<br>2. Cognitively able to follow instructions	Those individuals who had serious medical conditions, such as end-stage renal or heart disease that prohibited exercise, were refused from participa-tion.	Existing client database; Flyers; Posters; Word of mouth	Age: 77.2 (8.8)<br>Female %: 81.2<br>Black/African American: 251 (19.4)<br>Hispanic/Latino: 275 (21.2)	Not reported
Biedenweg (2014)	Older adults who had elected or declined to participate in physically activity-oriented program.	- Aged 50 years or older<br>- Resident of King County<br>- English fluency<br>- Able to understand the decision to participate	Not reported	Existing client database	Age: 73, 72, 69<br>Female %: 84 Joiner, 67 decliner, 88 quitter	Much less is known about the role of organized community-based programs that seek to support exercise

		- Enrolled in (or declined) the program at some point after January 2007 and before February 2008				behavior among older adults (Elward, Wagner, & Larson, 1992b). In particular, an understanding of older adult perceptions of the utility and appeal of such programs is largely lacking. This is especially true for older adults of diverse ethnic backgrounds and those who are socioeconomically disadvantaged (Conn et al., 2003) [p. 2].
Chiang (2008)	Ethnically diverse older adults participating in the EF exercise program in Seattle, Washington.	participation in the EF program for at least 1 month, being aged 55 or older, and the ability to read and speak English or Cantonese. p 3	Not reported	Existing client database	Age: 76.8 (7.4)<br>Female %: 85<br>Asian/Asian American: 24 (46)<br>Black/African American: 18 (35)	ethnic older adults
Fishleder (2019)	Older adults, primarily community-dwelling individuals aged 65 years and above who participate in the EF program	The data set consisted of EF program data collected regularly from January 2005 to June 2016. (p. 231)	First, we excluded participants who did not have any follow-up data after baseline (n = 14,050; 54.9%). There was also extreme variation in the time between the first and second physical function tests, ranging from 4 weeks to 11 years. Therefore, we excluded participants whose second physical function test date was more than 6 weeks deviant from the EF-defined program cycle (n = 4052; 15.8%). For example, we dropped participants with more	Not reported	Age: Most of the 7483 sample were between ages of 65 and 75 years (52%)<br>Female %: 81.3<br>American Indian/Alaska Native: 97 (1.3)<br>Asian/Asian American: 541 (7.2)<br>Black/African American: 881 (10.8)<br>Hawaiian Native/Pacific Islander: 26 (0.4)<br>Hispanic/Latino: 381 (5.1)	Not reported

			than 22 weeks between tests. Finally, because there was a large drop in attendance after the second program cycle, we included only the first 3 physical function tests. Put differently, sample participants had at least 2 test scores (baseline and the first program cycle) but may have had up to 3 test scores (a second program cycle). The final sample includes 7483 (29.2%) participants. Such heavy exclusions eliminated the possibility of analyzing the benefit of long-term involvement and may have introduced selection bias (discussed further in the Limitations section). To test for this bias, we compared the final sample with the excluded participants using logistic regression. (p. 231)			
Gell (2021)	Older adults interview - previously participated in the in-person EF classes (p.10)	Not reported	Not reported	Other: using a multimodal approach, including mailing letters and brochures to the UW Medicine patients and posting on social media.	Age: 74 (6.3)<br>Female %: Not reported; Woman %: 86<br>Black/African American: 3 (7)<br>Hispanic/Latino: 1 (2)	

Gell (2023)	Participants were recruited from two ongoing clinical trials (PACIFIC Study: NCT04099394 and JumpSTART: NCT04806139) that include Tele-EF classes (p. 972)	age 65 and older (PACIFIC) or 60 years and older (JumpSTART), not engaged in exercise prior to recruitment, English-speaking, community-dwelling, with symptomatic knee osteoarthritis and without cognitive impairment (PACIFIC) or a history of cancer and rural residence (JumpSTART)	Participants with atrial fibrillation or other arrhythmias were excluded from this study.	Other: existing clinical trials	Age: 72.2 (5.8)<br>Female %: Not reported; Woman %: 85.5%<br>American Indian/Alaska Native: 1 (1.8)<br>Asian/Asian American: 2 (3.6)<br>Black/African American: 2 (3.6)	The study included the rural residents. Inclusion criteria for the clinical trials were... community-dwelling, with symptomatic knee osteoarthritis and without cognitive impairment (PACIFIC) or a history of cancer and rural residence (JumpSTART).
Gell (2024)	Older rural cancer survivors	Eligible participants were age 60 or older, self-reported cancer survivors, as defined by Stage I–III cancer history with completion of adjuvant chemotherapy, radiation therapy, or surgery for their cancer diagnosis, and residing in a rural community, as indicated by zip code in RUCA areas designated as 4–10. Participants were not excluded on cancer type. Other criteria included minimal participation in exercise, confirmed with baseline accelerometer measures (< 150 min/week MVPA consistent with insufficient physical activity per Physical Activity Guidelines for Americans. (p. 597)	Among those screened, 46 (39%) were ineligible to participate based on inclusion criteria (e.g., no history of cancer, not living in a rural area, currently engaged in regular exercise). [T4, p. 5]<br><br>Additionally:<br>...one participant was deemed ineligible due to high physical activity levels as measured by the accelerometer. [T4, p. 5]	Flyers; Posters; Word of mouth	Age: 70.4 (5.7)<br>Female %: 79<br>American Indian/Alaska Native: 97 (1.2)	Yes, the study discusses equity considerations related to inclusion of underserved populations, specifically older rural cancer survivors.<br>Among those screened, 46 (39%) were ineligible to participate based on inclusion criteria (e.g., no history of cancer, not living in a rural area, currently engaged in regular exercise) ... Key to future implementation is the need for strategies to reach and recruit older rural cancer survivors who may not place a high value on exercise to participate in tele-exercise, particularly in situations where access is limited (pp. 5, 8)<br>The study references research on improving recruitment, sustainability, and scalability in physical activity programs for adults aged 50 years and older, including

						underserved populations (p. 10).
Gillette (2015)	Members of EF classes across the United States.	the individual had to have enrolled in an EF class that started between January 1, 2008 and June 16, 2008.	participants who lived in retirement communities and participated in a new EF class on-site due to differences in ease of access to EF classes	Other: postal mail solicitation from Senior Services, the organization that administers the EF program.	Age: 71.2 (8.23)<br>Female %: 89.2	Not reported
Greenwood-Hickman (2015)	Participants were members older than age 65 of GHC, which serves patients throughout the states of Washington and Northern Idaho. (p. 2)	Participants were selected by using the following eligibility criteria: Integrated Group Practice members (receiving medical care primarily within the GHC system), continuous enrollment for at least 1 year, aged 65 to 98, and eligible for the Medicare EF and SS programs for some portion of 2005 through 2011.<br>(p. 2)	Individuals were excluded if they met any of the following specific criteria: residing in long-term care or nursing home setting, receiving hospice care (International Classification of Diseases, 9th Revision, Clinical Modification [ICD-9-CM] code V66.7), wheelchair-bound (V46 or V53.8), aged 99 or older, or having a diagnosis of a serious mental health or substance use disorder (290–319.99, not including depression [296.2, 296.3, 300.4, 311], anxiety [300.02], or dementia [290]). (p. 2)	Not reported	Age: Nonuser: 74.1<br>Consistent: 73.7<br>Intermittent: 75.0<br><br>Female %: Nonuser: 55.6<br>Consistent: 73.7<br>Intermittent: 74.1<br><br>Asian/Asian American:<br>Consistent: 40 (7.9)<br>Intermittent: 96 (6.3)<br><br>Black/African American:<br>Consistent: 21 (4.2)<br>Intermittent: 60 (3.9)	Not reported
Jones (2024)	Sedentary/low-active adults with self-reported physician-diagnosed arthritis, across the adult age spectrum, under real-world conditions, in both urban and rural	Community-dwelling sedentary/low-active adults (60 min or less of self-reported, moderate-or vigorous-intensity, leisure-time PA per week;), aged 18 years or older, with self-	Not reported	Flyers; Posters; Word of mouth; Other: Participants were recruited by word-of-mouth, mail, e-mail, fax, door-to-door canvassing of	Age: 68.3 (10.6)<br>Female %: 86.1<br>Hispanic/Latino: 3 (1)	Recruiting rural adults was challenging due to rurality barriers and longer commutes (longest known commute was 45 minutes) (p. 9).<br>Of 672 screened, 437

	WV communities. (p. 2)	reported physician-diagnosed arthritis. (p. 2)		community businesses (e.g., pharmacies and barber shops), paid and nonpaid advertisements in newspapers and on the radio, and radio/television interviews with the Principal Investigator.		(65%) were eligible; 323 (74%) enrolled. Nonenrollees tended to be younger and from rural areas. Participants were mostly middle- and older-aged White women with high rates of chronic conditions, disability, and obesity (p. 4).
Kohn (2015)	Older adults participating in EF	Participated in EF between 2005 and 2011 in a community setting AND consented to have their information used for this research purpose.	Non-consenting. Missing data. Otherwise, the analyses focused on participants with baseline and follow-up data. (p. 3)	Not reported;<br>Other: [indicates that secondary data from 559 sites were extracted, thus each site likely had their own recruitment methods, which were not reported].	Age: 72.9 (9.8)<br>Female %: Not reported; Woman %: 83.1%<br>American Indian/Alaska Native: 291 (1.5)<br>Asian/Asian American: 846 (4.3)<br>Black/African American: 2823 (14.4)<br>Hispanic/Latino: 1373 (7)	Participants voluntarily enrolled and participated in EF classes available in their communities. Data were included only for participants who consented to share their information; 2,911 participants declined to share their information in this time period and are not included in the sample of 19,964. Participant information included: age, gender, race/ethnicity, marital status, education, household income, disability status, and health conditions. All demographics were voluntarily self-reported by participants at the time of enrollment in EF. (p. 3)
Kohn (2016)	Older adults who participated in the EF program between January 2005 and June 2012.	Eligible interview participants met the following criteria: participated in EF, taught EF, or oversaw EF in a staff position at a YMCA-affiliated location between January 2005 and June 2012	Not reported	Existing client database;<br>Other: recruiting letter and reminder recruiting postcards (p.307)	Age: 72.4 (11.9)<br>Female %: 85	Eligible EF participants were initially sampled purposively for maximum variation to represent diversity in geographic location, race, and household income based on the

		(as identified from administrative records); provided a complete mailing address; and consented to share their information for research purposes. (p. 307)				census tract in which their EF program operated. (p. 307)
Mays (2021)	Adults aged 50 and older who presented to the Cedars-Sinai Medical Network in Los Angeles, CA or self-referred themselves to the Health Coach from the surrounding community.	Aged 50 years or older, community dwelling, able to complete questionnaires, able to consent to participate in the study, and ability to communicate in English. (p. 3)	Participant were excluded if they had a known diagnosis of dementia, were permanent residents of nursing facility, or were unable to attend a community-based program. (p. 3)	Existing client database; Flyers; Posters; Other: Participants were recruited during outpatient visits with Cedars-Sinai Medical Network providers and self-referred from flyers distributed in physician offices, posted at the community host sites, and embedded in host site newsletters. Primary care physicians, subspecialist physicians, nurse practitioners, pharmacists, social workers, and case managers placed electronic referrals in the medical record. (p. 3)	Age: 76.8 (9.1)<br>Female %: 83.1	Not reported
Nguyen (2007)	Older adults with diabetes and they are members who were on the diabetes registry of GHC	Members who were on the diabetes registry of GHC, were not missing baseline cost and utilization data, and had at least 1 year of follow-up after the index date were included as EFP participants in this analysis (n = 163). (p44)	Not reported	Not reported	Age: 74.7 (5.2) Control<br>75.3 (6.0) EFP<br>Female %: EFP participants (n = 163): 66.9%; Control subjects (n = 364): 76.4%<br>Black/African American: 143 (37.9)	Not reported

Palmer (2016)	EF participants	Individuals over the age of 50 who were interested in participating in EF were enrolled by agencies as long as they were cognitively and physically able to participate in the EF program. (p. 2 - 2.2)	Exclusion criteria for the current study include (1) no data for the pretest, the posttest, or both; (2) a first FC that did not fall within 30 days before and seven days after the first attendance date (to assure that baseline data reflected actual abilities prior to any benefits received from attending EF sessions); (3) a second FC completed before the recommended dose was attained; and (4) more than six months between the first and second fitness checks	Not reported	Age: 74.8 years (SD ± 9.9)<br>Female %: N/A<br>Black/African American: 188<br>Hispanic/Latino: 677	1. Demographic characteristics of participants were self-identified on a form that included questions regarding gender, age, race and ethnicity, highest level of education, annual personal income, living situation (alone or with others), marital status, and primary spoken language. (p2)<br>2. The racial/ethnic composition of participants was diverse, with non-Hispanic whites and Hispanics making up the two largest groups. (p3)
Patel (2022)	Rural, community-dwelling older adults with symptomatic knee osteoarthritis	Inclusion criteria included (see Supplementary Material for screening questionnaire): age greater than or equal to 65 years, physician diagnosis of knee OA, symptomatic knee OA according to American College of Rheumatology criteria, knee pain–related difficulty with walking or going up and/or downstairs, community dwelling (not living in a nursing home), English speaking, and able to walk independently. (p. 736)	The exclusion criteria were cognitive impairment determined by a Mini Montreal Cognitive Assessment score of <11 (23,24) and any of the following in the past 6 months: cancer requiring treatment (except non-melanoma skin cancer), heart attack, stroke, hip fracture, hip and/or knee replacement, spinal surgery, heart surgery, deep vein thrombosis, or pulmonary embolus. (p. 736)	Existing client database; Flyers; Posters; Other: To reach the target population, we developed a tool kit for Arbor Health to promote the study, including a study brochure, poster, draft letter to patients identified through electronic health records, and content for print advertisements and social media postings. Arbor Health used all components of the tool kit to promote	Age: 71.8 (5.8)<br>Female %: Not reported; Woman %: 93.3	1. Study participants were residents of Lewis County, Washington, which is a rural county based on the CDC's National Center for Health Statistics urban–rural classification. (p. 736)<br><br>2. Access to evidence-based exercise programs is severely limited in rural areas. Many rural residents are unable to participate in community-based exercise because of limited or lack of access to transportation and exercise facilities.

				the project, and the research team at the UW fielded inquiries and screened potential participants by telephone. Recruitment occurred between December 18, 2020, and March 16, 2021. Verbal informed consent was obtained from all study participants. Compensation for participating in the study was $100. (p. 736)		Walking is a common choice for exercise among older adults, yet walking in rural areas is limited by lack of pedestrian infrastructure, including long distances between destinations and lack of sidewalks. Considering that rural communities have a higher burden of arthritis and obesity, as well as a higher proportion of older adults, than nonrural areas, there is a critical need to adapt evidence-based exercise programs for remote delivery. (p. 735)<br><br>3. Participants had multiple chronic conditions, moderate-severe pain and functional limitations, and were at high risk for falls and mobility disability.<br><br>4. The CDC and other groups have recognized the need to improve equity in access to and delivery of evidence-based OA management program. (p. 741)<br><br>5. Indeed, our partnership with Arbor Health was critical to reaching the current study's target population. Anecdotally, some participants shared that

						receiving the recruitment letter from Arbor Health and checking with their physician helped assure them about the legitimacy of the study and tele-EF program. (p. 742)
Rosenberg (2014)	Participants were between ages 65 and 99 and were continuously enrolled Medicare members of GH from January 1, 2009, through December 31, 2011.	Participants were between ages 65 and 99 and were continuously enrolled Medicare members of GH from January 1, 2009, through December 31, 2011. (p. 246)	1. We excluded people residing in long-term care, hospice care, or skilled nursing facilities; who were wheelchair-bound; or who had serious mental health or substance use disorders (not including depression, anxiety, or dementia). (p. 246)<br><br>2. Those without a visit during 2009-2010 were excluded from the analysis. We compared users of SS and users of EF with nonusers of either program. (p. 247)	Existing client database; Word of mouth; Other: Websites, mailing	Age: 76.4 (7.5)<br>Female %: 74%<br>Hispanic/Latino: 21 (2.8)	1. Of eligible to use EF, 1.9% used EF in 2011.<br><br>2. EF users were generally older and less likely to be male in adjusted models than non-users (p. 247)<br><br>3. EF users had significantly lower Charlson score (comorbidity score) than non-users.
Sin (2005)	Elderly Korean immigrants	(a) ≥60 years old; (b) able to read, speak, and write Korean; (c) written permission from a physician to participate in this program; and (d) have all measurement data (focus group and health outcomes).	Not reported	Flyers; Other: help from a Korean social worker in the senior house. (p409)	Age: 77 (5.9)<br>Female %: 61.5<br>Asian/Asian American: 13 (100)	All exercise participants were residents of the senior house. EKIs were recruited through fliers posted in a senior house and help from a Korean social worker in the senior house.<br>The senior house was built mainly for the low-income, minority elderly and was located in downtown Seattle.

Smith (2014)	EF participants from Arizona, Florida, Hawaii, Iowa, Maine, Michigan, Minnesota, Oklahoma, Oregon, and Texas.	EF participants during 2006-2009 from Arizona, Florida, Hawaii, Iowa, Maine, Michigan, Minnesota, Oklahoma, Oregon, and Texas.	Not reported	Existing client database; Other: Strategies: focus groups, advertisements, announcements, and short stories using case examples in community newspapers, Use testimonials and word of mouth, post testimimonials available on Website, Recruit bi-lingual (eg, English and Spanish) instructors, Produce EF promotional materials and data forms in the language of participants,	Age: 71.38 (±10.65)<br>Female %: 84.2<br>Black/African American: 957 (19.9)<br>Hispanic/Latino: 585 (12.1)	KAEA now offers the program in 4 of the 5 districts of the island. Fully 80% of the Kauai EF participants are Asian American and Pacific Islander.<br>(p. 153)<br>Nearly half of the participants were from racial/ethnic minority backgrounds and were more complex and frail patients with increased pre-existing conditions upon enrollment.
Steinman (2024)	Sampling prioritized organizations engaging older populations with low-income status with multiple chronic conditions who are vulnerable to both COVID-19 infection and poor health outcomes and care (eg, people of color, those with disabilities, and those living in rural areas). (p. 3)	We used maximum variation purposive sampling [25] to identify organizations of diverse sizes and types, varied provider characteristics, and from different geographic areas to aid the generalizability of the evaluation findings. This sampling prioritized organizations engaging older populations with low-income status with multiple chronic conditions who are vulnerable to both COVID-19 infection and poor health outcomes and care (eg, people of color, those with disabilities, and those living in rural areas). The eligibility criteria for	Not reported	Existing client database	Age: 72.2 (8.5)<br>Female %: 100<br>Asian/Asian American: 2 (16.7)<br>Black/African American: 1 (8.3)<br>Hispanic/Latino: 1 (8.3)	We used the RE-AIM (Reach, Effectiveness, Adoption, Implementation, and Maintenance) for equity framework to evaluate the potential impact of remote EBPs on older adults' health and well-being. The equity lens means that in addition to evaluating impact, we looked at outcomes across programs, sampled organizations that reach older adults who are underserved, reported EBP reach, and assessed whether there were any unintended consequences. As such,

		organizations was the delivery of at least 1 of the 5 remote EBPs from January 2021 through March 2022. We then used convenience sampling to invite all remote EBP participants to take part in the evaluation. (p. 3-4)				we used mixed methods to give voice to participants as well as report statistical trends. (p. 1)
Sugihara (2011)	Adults age 60 and older on Kaua'i	Not reported	Not reported	Other: Recruitment presentations were conducted at the first two centers, Koloa and Waimea Senior Centers, located 14 miles apart. Recruitment packets were developed that included registration, health history, and consent forms. (p. 117)	Age: 80 years<br>Female %: 96.3%	Not reported
Tomioka (2012)	Rural community-dwelling older adults of Kaua'i Hawai'i who were Asian/Native Hawaiian/Pacific Islander (API) or white.	Rural community-dwelling older adults of Kaua'i Hawai'i who were Asian/Native Hawaiian/Pacific Islander (API) or white who enrolled in EF between 2007 and 2010.	Not reported	Other: The survey asked how program participants heard about EnhanceFitness (county agency, service providers, doctors/health care professionals, mass media [newspaper, brochure, television, or radio], or other (p. 3)	Age: 78.2 (no SD given) range was 61-95<br>Female %: 93.7<br>Hawaiian Native/Pacific Islander: 17 (17.6)	Kaua`i County has approximately 13,800 (21%) older adults. Of these older adults, 53% are Asian/Pacific Islander (API), 34% report at least 1 disability, and 60% experience loss of physical performance. Kaua`i County is rural and is challenged by limited health promotion opportunities for older adults (p. 2)
Tomioka (2019)	Participants ages 50+ who participated in EF in Hawai'i.	50+ who attended EF in 19 sites from 2007 to 2016.	No elder was excluded from joining the program, and informed consent was obtained from all participants (p. 2) Cases were omitted	Existing client database; Word of mouth; Other: [recruited over time through Area Agencies on Aging and their Aging and	Age: Not reported<br>Female %: 90.2%<br>Asian/Asian American:<br>Filipino: 46 (10.8)<br>Japanese: 198 (46.4)<br>Hawaiian	diverse older adult population of Caucasians, Asians, Native Hawaiian, other Pacific Islanders, and others. To ensure the attractiveness of and

			for missing data. Only participants with continuous enrollment for 12 mos who participated in 4-, 8-, and 12-month Fitness Checks	Disability Resource Centers (28%), eldercare, faith-based, and healthcare organizations (31%), word-of-mouth (15%), health professionals (2%), and other avenues including community events and mass media (24%).]	Native/Pacific Islander: 32 (7.5)	practicality of EF for Hawai'i older adults, HHAP worked with the program developers to make minor modifications, such as renaming certain exercises to relate them to daily activity and utilizing culturally appropriate music.
Tomioka (2024)	Older adult participants in EnhanceFitness in Hawaii	Participants from a pool of 493 Enhance®Fitness active members who enrolled in the program between April 2019 and March 2020. (p. 181)	Not reported	Existing client database	Age: 74<br>Female %: 94%<br>Hawaiian Native/Pacific Islander: 32 (11.1)	Not reported
Wallace (1998)	Older adults aged 65 years and older who are ambulatory and living in the catchment area of the Northshore Senior Center	Subjects were eligible if they were age 65 or over and ambulatory. Potential subjects were invited to the senior center for screening tests and a brief evaluation by a study physician in order to identify individuals too disabled, too cognitively impaired, or too ill to participate in the trial. Specific exclusion criteria included legal blindness; a timed 'Up and Go' test (9) greater than 30 s ...; a score of less than 24 on the Folstein Mini-Mental State exam (10); a myocardial infarction or change in angina pattern in the past year; presence of other medical conditions that precluded or contraindicated exercise (i.e., end-stage heart or lung disease, recent	Specific exclusion criteria included legal blindness; a timed 'Up and Go' test (9) greater than 30 s ...; a score of less than 24 on the Folstein Mini-Mental State exam (10); a myocardial infarction or change in angina pattern in the past year; presence of other medical conditions that precluded or contraindicated exercise (i.e., end-stage heart or lung disease, recent deep venous thrombosis, severe degenerative joint disease requiring joint replacement, severe inflammatory arthritis). In addition,	Flyers; Other: Newsletter	Age: 71.9<br>Female %: 73	Concerted efforts will be needed to attract less educated, less affluent individuals with less healthy lifestyles to senior centers, and to their health-related activities. Although some success in attracting frailer, at-risk elders to senior centers for health promotion activities has been demonstrated, it is likely that some high-risk populations will require alternative strategies (e.g., proactive phone follow-up or home-based programs) to access and engage in disability-prevention interventions (p. 305)

		deep venous thrombosis, severe degenerative joint disease requiring joint replacement, severe inflammatory arthritis). (p. 302)	each subject, primary physician was contacted to ascertain if he or she had concerns about the participation. (p. 302)			

Table S3. Adoption

Study	Geographical location of intervention	Adoption type (organization/ community center/church/other)	Number of sites participated	Characteristics of EF instructor reported
Ackermann (2003)	Western Washington state	Organization	Not reported	No
Ackermann (2008)	Seattle/Puget Sound area in Washington State	Organization	Not reported	No
Agmon (2015)	Not reported	Other: in various community locations	Not reported	No
Batra (2016)	Broward, Miami-Dade, and Monroe counties in South Florida.	Organization; Community center; Church; Other: Sites chosen by the agencies included adult day care centers, senior centers, clinics, hospitals, skilled nursing facilities, assisted living facilities, community centers, or senior housing. (page 650)	160 workshops but not sure how many sites that represents	Yes<br>Classes were led by certified instructors. An initial group of HARC EF Master Trainers were trained by T-Trainers from Senior Services, an organization that oversees the management of EF. HARC Master Trainers were then held responsible for training HARC EF instructors who led EF classes. All the trainings were conducted using the EF instructor's manual. Each provider agency and its partner agencies identified Master Trainers and EF instructors. In addition to EF-specific training, all Master Trainers and EF instructors were required to be certified by a nationally recognized fitness organization such as the American Council on Exercise, American College of Sports Medicine, or Young Men's Christian Association. To ensure fidelity, all newly-trained instructors taught their first class under the supervision of a Master Trainer. (page 650)
Batra (2019)	three southeast counties of South Florida: Broward, Miami–Dade, and Monroe (p. 402)	Community center; Other: adult day care centers, senior centers, clinics, hospitals, skilled nursing facilities, assisted living facilities, or senior housing	14	Yes<br>Potential instructors were recruited from within partner agencies or were identified using a variety of external methods, including word of mouth, flyers, and advertisements. To be considered eligible for an instructor, an individual was required to have certain qualities such as good communication and interpersonal skills, enthusiasm and readiness to lead a small group of older adults, ability to carry up to 20 pounds weight, and ability to perform a range of motions and low-level endurance exercises. An EF master trainer from Senior Services, an organization that oversees the management of EF, trained all EF instructors. In addition, all instructors were required to have a fitness certification

				from a nationally recognized certifying organization.(page 403)
Belza (2015)	The intervention took place in multiple locations across the United States, specifically involving 10 different YMCA associations in six states. The exact states are not listed in the provided text, but staff were from varied locations representing these associations.	Community center	14	Yes<br>Staff mentioned factors such as instructor ownership of the class and the match between instructor and participant characteristics as elements influencing class success or barriers [p. 4].
Biedenweg (2014)	King County, Washington	Church; Other: Senior center	2	No
Chiang (2008)	Seattle, WA	Community center; Church; Other: Senior centers	3	No
Fishleder (2019)	41 states and Wash DC	Community center	Not reported	Yes<br>Classes are taught by certified instructors. Project Enhance strongly recommends that affiliate organizations' instructor candidates have a primary fitness certification or experience in physical therapy, occupational therapy, or nursing before attending the 12-hour in-person EF New Instructor Training certification. EnhanceFitness certification includes 3 pretraining modules and a 1.5-day in-person certification. EnhanceFitness master trainers support EF instructors through new instructor training, mentoring, and fidelity monitoring. Prerequisites to EF master trainer training include 1-year teaching EF to fidelity and a National Commission for Certifying Agencies-accredited fitness certification or degree. Continuing education for all EF instructors titled Essentials for EF Instructors is available through Project Enhance's American Council on Exercise portal (http://www.projectenhance.org/EnhanceFitness/Enact.aspx), available online only to certified EF instructors. The purpose

				of continuing education is to support the continued development of skills and maintain certification. (page 231)
Gell (2021)	Seattle, Washington	Other: Academic	1 site (remote): 3 cohorts	Yes<br>The tele–EnhanceFitness classes were led by EnhanceFitness-certified instructors. (p2)
Gell (2023)	Not reported	Not reported	Not reported	Yes<br>Instructors for EF are certified by the American Council on Exercise and complete training with an EF Master Trainer that includes teach-back demonstration of exercises according to program protocols, including exercise modifications (e.g., seated exercise) to accommodate older adults with different levels of fitness and functioning. (p. 973)... Instructors were in the same relative geographic location as their participants.
Gell (2024)	Conducted with participants residing in rural communities as indicated by RUCA codes 4–10.	Community center	Not reported	Yes<br>Instructors are certified by the American Council on Exercise and undergo 12 hours of training with an EF Master Trainer, which includes audiovisual materials, live demonstration, and teach-backs to ensure understanding of program protocols and exercise modifications, including seated options. They demonstrate two levels of each exercise to accommodate participants' functional abilities and symptoms.
Gillette (2015)	Midwest, West, and Northeast regions of the United States.<br>No specific states were mentioned.	Not reported	17	
Greenwood-Hickman (2015)	Seattle, Washington	Not reported	Not reported	
Jones (2024)	Urban and rural West Virginia	Community center; Church; Other: A rehabilitation center and a recreational center	Eighteen exercise sites were invited to join the study (13 senior centers, three churches, one rehabilitation center, and one recreational center). Seventeen (94%) of 18 exercise sites joined the	Yes<br>All 17 exercise sites had a trained instructor and began EF for a site adoption rate of 100%. Instructors were fitness instructors, senior center staff, community members, extension agents, or aging services staff. Of 21 invited instructors, 10 (48%) underwent the YMCA fitness certification and 20 (95%) underwent EF training. Nine (45%) of the 20 instructors withdrew (three job-related, three unknowns, one moved, one started school, one due to health concerns). Six (67%) of those nine instructors were beginners

			study. One (6%) senior center declined (administrative reasons). All 17 exercise sites had a trained instructor and began EF for a site adoption rate of 100%. (page 4)	(no experience/less than 2 years of experience), 1 (11%) was intermediate (taught at least 1 class in past 2 years), and 2 (22%) were experienced primary occupation in fitness field). Eleven (8 females, 3 males, aged 30 to 69) of 20 trained instructors started EF for an instructor adoption rate of 55%. Two (18%) of the 11 instructors were beginners, 4 (36%) were intermediate, and 5 (46%) were experienced. (page 4)
Kohn (2015)	Nationwide: secondary data from 559 sites were extracted from Senior Services	Organization; Community center; Church; Other: Senior centers, social service organizations, residential sites, recreational organizations, healthcare organizations, faith-based organizations, YMCAs, and other sites	559	No
Kohn (2016)	Not reported	Not reported	Not reported	No
Mays (2021)	9 sites surrounding Cedars-Sinai Geriatrics Program in Los Angeles, CA.	Organization; Community center	9	Yes<br>All four EBPs (Table 1) were taught by instructors from two community-based nonprofits – Jewish Family Service and Partners in Care with experience in running EBPs. Study classes and the program community health coach were funded through the AARP Foundation. All instructors were certified to teach and follow the guidelines for each class as outlined in the prior clinical trials during which the classes were developed (page 4).
Nguyen (2007)	Not reported	Not reported	Not reported	Yes. The program, taught by certified fitness instructors
Page (2014)	South Florida	Community center	7	Yes<br>Costs of instructors was discussed. Forty-three percent of costs went toward class instructors. Training costs were paid for by HARC and, therefore, not included in the analysis of agency costs. Differences in costs across Year 2 agencies are because of different compensation for instructors. Agency 4 was able to reduce costs by paying volunteer leaders $8 to $13 per hour to conduct classes. Agency 3's costs were

				higher than other second-year agencies because it paid $52 per hour for instructors. Agency 6 achieved lower costs by spending only $9,080 per year on program administration. Agency 7 did not have any costs reported for class instructors. A full-time employee was hired to handle program administration and participate in class instruction. The agency could not report what percentage of the employee's effort was devoted to each task, so the $40,000 listed under "administrative costs" also includes that employee's participation in delivering the classes. They also recruited two unpaid volunteers to serve as class instructors. (page 589)<br><br>Of note: One recommendation: recruiting volunteer or low-cost instructors was important in achieving lower monthly costs. (page 590) yet no mention of the quality of instruction if instructors are paid less.
Palmer (2016)	South Florida	Organization; Community center; Church; Other: senior housing, senior centers, and parks	83	Yes<br>Instructors who were certified by a nationally recognized fitness organization (i.e., YMCA, ACE, or ACSM) and who received training in EF procedures at a two-day training conducted by EF program developers and EF master trainers led classes. All instructors were required to teach EF as outlined in EF training materials provided by the program developer. Individuals over the age of 50 who were interested in participating in EF were enrolled by agencies as long as they were cognitively and physically able to participate in the EF program.<br>(p2)
Patel (2022)	Lewis County (rural) in Washington state	Organization	1	Yes<br>Experienced, EF-certified instructor
Petrescu-Prahova (2016)	Not reported, but from a national sample of trainers and staff	Community center; Other: All sites were YMCA affiliated	Not reported (but 15 instructors and 15 staff participation)	Yes<br>I don't think that [EF instructors] have enough knowledge after a two-day training to take on the responsibility of people's health (p. 4)<br>Entire paper is about EF instructors (n=15), Master Trainers (n=2), and program staff (n=15) affiliated with the YMCA
Petrescu-Prahova (2022)	13 states in the US	Organization	20	Yes<br>20 of 21 sites were eligible. 20 of 20 (100% joined the intervention)

Rosenberg (2014)	Western Washington state	Organization	N/A	Yes<br>Instructors are certified to teach the program through a licensing process administered by Senior Services of Seattle. (page 2)
Sin (2005)	Seattle, Washington; The senior house was built mainly for the low-income, minority elderly and was located in downtown Seattle. (p. 408)	Other: senior house	1	Yes<br>All instructors are required to hold a nationally recognized fitness instructor certification and attend the LFP instructor training before taking classes. The LFP© includes all four components of activity felt to be important for seniors—balance, flexibility, strength training, and aerobic capacity. The bilingual investigator was born and raised in Korea and hence was familiar with Korean language and culture. (p409)
Smith (2014)	Arizona, Florida, Hawaii, Iowa, Maine, Michigan, Minnesota, Oklahoma, Oregon, and Texas	Organization; Community center; Church; Other: health care organization, Faith-based Organization, Recreational Organization, Senior Center, Multi-purpose Social Services Organization, Other Community Center, Parks Department Facility, Residential Facility, Other	No number:<br>senior centers (26.6%) and multi-purpose social services organizations (23.7%) followed by other community centers (12.0%), healthcare facilities (11.8%), residential facilities (10.0%), other sites (7.3%), and faith-based organizations (6.5%).	Yes<br>"These staff members provided a wide array of assistance at local and regional levels, including trainings for EF Master Trainers and instructors, leadership trainings on PRE-AIM (Planning, Reach, Effectiveness, Adoption, Implementation, Maintenance) principles, coalition building, asset inventory, gap analysis, funding for startup costs for EF licenses and weights, technical assistance, and hosting of annual EF leader and master trainer workshops and conferences."<br>(Page 152)<br><br>"Many of the trained EF instructors who led workshops were bilingual in both English and Spanish and many of the EF classes had a mix of both English and Spanish speaking participants"<br>(Page 155)
Steinman (2024)	USA (33 states)	Organization; Community center	Not reported	No
Sugihara (2011)	Kaua'i, Hawaii	Organization; Community center	1	Yes<br>staff and trainers from Senior Services; 1 Master Trainer and 7 EF instructors)
Tomioka (2012)	Kaua'i county in the state of Hawai'i	Community center	8 classes at 7 sites	No
Tomioka (2019)	19 EF sites in Hawai'i	Organization; Community center; Church; Other: (8 multipurpose centers, 4	19	No

		senior housing facilities, 5 faith-based organizations, 1 adult daycare, and 1 fitness gym)		
Tomioka (2024)	Hawaii	Community center	2 (2 counties: Maui and Kaua'i)	No
Wallace (1998)	Seattle King County	Community center	1	No

Table S4. Maintenance

Study	Long-term effects	Continued delivery reported (Yes/No)	Cost of maintenance reported (Yes/No)
Ackermann (2003)	post intervention	Yes	No
Ackermann (2008)	0–12 months (Year 1), 13–24 months (Year 2)	Yes<br>Since October 1998, GHC has paid the per-visit costs for all of its Medicare-eligible enrollees who elect to participate in the EF program. p.1460	No
Agmon (2015)	post intervention	No	No
Batra (2016)	post 4 months	Yes<br>(page 655) HARC offered EF on an ongoing basis in South Florida, and among the 75% that did not meet the completer definition criteria, 36.7% attended more than 32 sessions in total but were not considered a completer because they did not complete those within 4 months from their first date of attendance.	No
Batra (2019)	4-month, 8-month, and 12 month	Yes<br>HARC offered EF on an ongoing basis. (p403)	No
Belza (2015)	No	No	No
Biedenweg (2014)	No	No	No
Chiang (2008)	No	No	No
Fishleder (2019)	8 months	No	No
Gell (2021)	No	No	No
Gell (2023)	post intervention	No	No
Gell (2024)	16 weeks (post-intervention)	No	No
Gillette (2015)	No	Yes<br>Current participants must be in the class.	No
Greenwood-Hickman (2015)	6 years	No	No
Jones (2024)	12 weeks	Yes<br>Only three sites and instructors continued EF even though they were encouraged to continue, EF licenses were active, and equipment was left at the sites. The high rate of discontinuation was most likely due to a lack of instructor funding, a known barrier to maintenance. (page 10)	No
Kohn (2015)	4-month	No	No
Kohn (2016)	No	Yes<br>“At the time of the interviews, 9 (45%) participants were currently participating in EF and 11 (55%) were no longer	No

		participating in EF.<br>(Page 308)	
Mays (2021)	8 weeks (for EF), & 6 months	Yes<br>In May 2020 our classes transitioned to being offered virtually via Zoom for both Arthritis Exercise and Tai Chi for Arthritis with future analyses to examine the transition from in-person to virtual programming. (page 11)	No
Nguyen (2007)	12-month	Yes<br>"The EFP is a group-based exercise program that meets 3 times/week and is currently offered to community-dwelling older adults at ~30 community-based sites in the Seattle/Puget Sound area.<br>(p44)	No
Page (2014)		Yes<br>(page 587) The goal is for HARC to cover program start-up costs, and once the HARC initiative ends in 2014 the agencies will continue to offer classes on their own.	Yes<br>Average monthly costs per class were $1,713 during the first year of implementation and $873 during the second year of implementation. The cost measurements, combined with information from the literature on cost savings attributable to EnhanceFitness participation, suggest that EnhanceFitness has the potential to generate a net societal cost savings among program participants. The results are useful for community agencies considering implementing EnhanceFitness for their populations. (page 585)
Palmer (2016)	16 weeks	Yes<br>At the end of the three-year period, nine agencies were funded and active, offering 70 ongoing EF classes at 56 sites (p3)	No
Patel (2022)	12 weeks	Yes<br>Partnerships with health care systems can also help sustain programs. Arbor Health, for instance, obtained an EF license and is now offering tele-EF classes on the basis of feedback received from study participants. (page 742)	No
Petrescu-Prahova (2016)	No	No	No
Petrescu-Prahova (2022)	After 30 month trial period	Yes	No

Rosenberg (2014)	No	Yes<br>Ongoing program	No
Sin (2005)	12 weeks	No	No
Smith (2014)	Over a 3 year period (2006-2009)	Yes<br>To support ongoing maintenance of the program, EF was integrated as 'usual service' for the AAA and service providers.<br>(Page 153)	Yes<br>Findings from this study highlight the capacity of states to reach diverse populations through broad-based techni cal assistance, infrastructure development, creative funding, and recruitment and retention strategies salient to local communities of older adults. (p. 157)
Steinman (2024)	6 months	No	No
Sugihara (2011)	12 months	Yes	Yes<br>The total cost of the program to operate six sites, based on operational costs of the first site, was $180,476 in 2004. Adjusting to 2009 with a 2.6% average rate of inflation,17 the cost would have been $204,735. Recurrent costs were 92% of the budget and the 8%, one time, up front costs, are listed in lines 1 and 3 of Table 2. Although instructor training, line 4, might seem to be a one time cost, a conservative estimate accounts for high turnover of instructors and tallies these expenses as ongoing. An itemized list is presented in Table 2. (page 118)
Tomioka (2012)	4 months	Yes<br>119 (53%) of participants attended longer than 1 year.	No
Tomioka (2019)	4 months, 8 months, 12 months	No	No
Tomioka (2024)	No	No	No
Wallace (1998)	6 months	Yes<br>After the 6-month trial period ended, 51% of controls joined the supervised exercise program and about half of intervention subjects continued to attend the exercise classes (p. 303)	No
